# Innovative technology‐based interventions in Parkinson's disease: A systematic review and meta‐analysis

**DOI:** 10.1002/acn3.52160

**Published:** 2024-09-05

**Authors:** Chun En Yau, Eric Chi Kiat Ho, Natasha Yixuan Ong, Clifton Joon Keong Loh, Aaron Shengting Mai, Eng‐King Tan

**Affiliations:** ^1^ Yong Loo Lin School of Medicine National University of Singapore Singapore Singapore; ^2^ Department of Neurology Singapore General Hospital Campus, National Neuroscience Institute Singapore Singapore; ^3^ Neuroscience and Behavioural Disorders Duke‐NUS Medical School Singapore Singapore

## Abstract

**Objective:**

Novel technology‐based interventions have the potential to improve motor symptoms and gait in Parkinson's disease (PD). Promising treatments include virtual‐reality (VR) training, robotic assistance, and biofeedback. Their effectiveness remains unclear, and thus, we conducted a Bayesian network meta‐analysis.

**Methods:**

We searched the Medline, Embase, Cochrane CENTRAL, and Clinicaltrials.gov databases until 2 April 2024 and only included randomized controlled trials. Outcomes included changes in UPDRS‐III/MDS‐UPDRS‐III score, stride length, 10‐meter walk test (10MWT), timed up‐and‐go (TUG) test, balance scale scores and quality‐of‐life (QoL) scores. Results were reported as mean differences (MD) or standardized mean differences (SMD), with 95% credible intervals (95% CrI).

**Results:**

Fifty‐one randomized controlled trials with 2095 patients were included. For UPDRS (motor outcome), all interventions had similar efficacies. VR intervention was the most effective in improving TUG compared with control (MD: −4.36, 95% CrI: −8.57, −0.35), outperforming robotic, exercise, and proprioceptive interventions. Proprioceptive intervention significantly improved stride length compared to control intervention (MD: 0.11 m, 95% CrI: 0.03, 0.19), outperforming VR, robotic and exercise interventions. Virtual reality improved balance scale scores significantly compared to exercise intervention (SMD: 0.75, 95% CrI: 0.12, 1.39) and control intervention (SMD: 1.42, 95% CrI: 0.06, 2.77). Virtual reality intervention significantly improved QoL scores compared to control intervention (SMD: −0.95, 95% CrI: −1.43, −0.52), outperforming Internet‐based interventions.

**Interpretation:**

VR‐based and proprioceptive interventions were the most promising interventions, consistently ranking as the top treatment choices for most outcomes. Their use in clinical practice could be helpful in managing motor symptoms and QoL in PD.

## Introduction

With the fast aging of the world's population, the prevalence of Parkinson's disease (PD),^1^, ^2^ a neurodegenerative condition, will increase significantly. In 2016, it was estimated that the 6.1 million people live with PD globally.^3^ From 1990 to 2016, the age‐standardized rate of prevalence had increased by 22%, and age‐standardized change in disability‐adjusted life years has also increased.^4^ PD patients present with bradykinesia, defined as slowness of movement and reduction in amplitude or speed as movements, are continued, in combination with either rest tremor, rigidity, or both.^5^, ^6^, ^7^, ^8^ Postural instability often present later in the course of the disease. Such symptoms vastly affect patients' motor ability and their quality of life (QoL).^9^, ^10^

There is increasing interest in motor‐cognitive rehabilitation therapies for PD patients but their effectiveness remains to be clarified.^11^ Pharmacologic therapies including dopaminergic drugs such as levodopa and dopamine agonists^2^ are frequently associated with long‐term complications such as dyskinesias and motor fluctuations^2^, ^6^ and can also aggravate nonmotor parkinsonian symptoms such as hallucinations, cognitive impairment, and orthostatic hypotension. Several motor features, including gait and balance, do not typically respond to levodopa.^12^ Another common treatment is deep brain stimulation, which involves sending electrical signals to specific brain nuclei. While it has been proven effective in improving quality of life and “appendicular” motor symptoms such as limb tremor, it remains insufficient in reducing axial motor symptoms such as gait impairment and postural abnormalities^13^ and has been shown to worsen speech intelligibility.^14^

Balance and gait are motor outcomes that are frequently affected in PD. These alterations are often associated with diminished functional ability, poor prognosis, and frequent falls.^15^ To supplement pharmacological and surgical treatment, there has been an increasing focus on using technology‐based interventions to improve motor outcomes and quality of life.^16^, ^17^, ^18^, ^19^ Virtual‐reality (VR) training has been effective in improving motor function in chronic stroke patients.^20^ VR technology can provide PD patients with a safe and effective environment to undergo training and rehabilitation.^16^ Rehabilitation assisted by robotic machines also serve to help patients achieve better motor outcomes by acting as a force multiplier for conventional physiotherapy and training.^17^ Rehabilitation approaches such as peripheral stimulation are noninvasive, and they show potential in improving motor outcomes.^18^ Internet‐based interventions are also of note as they improve access to care for patients,^19^ hence allowing for better follow‐up with patients that can contribute to positive outcomes. As motor functions may be influenced by one's cognitive ability,^21^ interventions that integrate motor and cognitive aspects have the potential to lead to better motor outcomes in rehabilitation.^22^, ^23^, ^24^ Technology‐based interventions, such as the addition of a VR component to treadmill training, show promise in delivering outcomes in both motor and cognitive domains through an integrated motor‐cognitive approach.^25^ Therefore, these technology‐based interventions may be used to complement ongoing pharmacological or surgical treatment and improve rehabilitation outcomes for patients.

However, for technology‐based interventions, there are few large‐scale randomized controlled trials evaluating the effectiveness of available interventions, and very few studies have directly compared the interventions against each other. Randomized controlled trials comparing several treatments are usually not feasible^26^ due to sample size limitations, and conclusions concerning the effectiveness of these interventions are varied. To our knowledge, there is no existing study that directly compares the effects of these interventions. To address this gap in knowledge, we conducted a network meta‐analysis, which allows for direct and indirect comparison^26^ among Internet‐based, proprioceptive, robotic, and VR interventions against active and inactive controls, and their effects on QoL, gait, and balance parameters. For this study, Internet‐based interventions were defined as any form of telemedicine, or any intervention which involved the input of healthcare professionals remotely via online means. Proprioceptive interventions were defined as any intervention that increased the proprioceptive input to the patient (e.g., through mechanical pressure stimulations or vibrations). Robotic interventions, inclusive of gait and balance training assistance, utilized robotic means to facilitate sensorimotor rehabilitation to the patients.^27^ Virtual reality interventions generate an environment in which the patient can interact in a manner that is similar to a physical place.^28^

With improved understanding of the impact of these interventions, the potential of these interventions can be better maximized to improve overall rehabilitation outcomes.

## Methods

### Search strategy

This network meta‐analysis was conducted following the Preferred Reporting Items for Systematic Reviews and Meta‐Analyses (PRISMA) guidelines (PROSPERO CRD42022301160).^29^ The PRISMA checklist can be found in Supplementary Material S1. The Medline, Embase, Cochrane CENTRAL, and Clinicaltrials.gov databases were searched from inception until 2 April 2024. The search strategy involved keywords and MeSH terms synonymous to “Parkinson Disease”, “robotics”, “internet‐based”, “virtual reality”, “biofeedback”, “physical stimulation”, “exergaming”, and “rehabilitation”. References of related reviews were screened to ensure a comprehensive search. A copy of the search strategies can be found in Supplementary Material – Table S1.

### Study selection and data extraction

Four independent authors (CEY, ECKH, NYO, and CJKL) carried out the eligibility assessment in an independent and blinded manner. The authors screened the titles and abstracts before retrieving and reviewing the full texts. A third independent author (ASM) was involved in the resolution of disputes. Only randomized controlled trials with more than 10 participants in each arm were included. Observational studies, case–control studies, reviews, meta‐analyses, editorials, commentaries, conference abstracts, and non‐English language articles were excluded. Studies were included if they (i) were randomized controlled trials that (ii) evaluated outcomes related to balance and gait (iii) in patients with PD.

The primary study outcomes were changes in measures for UPDRS (motor outcome), stride length, 10‐meter walk test (10MWT), timed up‐and‐go (TUG) test, balance scale scores, and QoL indices.

All controls for the studies, excluding the proprioceptive studies, were either usual care or active controls, where participants engaged in exercise or conventional physical therapy. For three‐armed studies with two active controls, the more common active control was chosen. For three‐armed studies with an active control and usual care, all three arms were included. For the proprioceptive studies, controls were placebos. For the reporting of the results, active controls are labeled “Exercise,” while usual care/placebo are labeled “Control.”

Data were extracted by four authors (YCE, ECKH, NYO, and CJKL) in an independent and blinded manner. The following variables were extracted: (i) baseline demographics: age, gender, disease duration, MDS‐UPDRS/UPDRS Section III scores and (ii) changes in MDS‐UPDRS/UPDRS Section III score, stride length, 10MWT, TUG test, balance scale scores (e.g., Mini Balance Evaluation Systems Test scores and Parkinson Disease Questionnaire Scores), and Quality of Life scores (e.g., SF‐36 and PDQ‐39).

### Statistical analysis

All analyses were conducted in RStudio (Version 4.0.3). The Bayesian network meta‐analysis was performed with the *BUGSnet* package. Mean differences (MD), standardized mean differences (SMD), and 95% credible intervals (95% CrI) were used. MD was calculated for stride length, 10MWT, and TUG test as these were continuous outcomes. A variety of scales were used for the outcomes of UPDRS (motor outcome), balance, and QoL scores. Hence for these outcomes, we calculated SMD, which is often employed to compare and meta‐analyse heterogenous measures of an outcome.^30^, ^31^ Treatment groups were namely (i) Internet‐based, (ii) proprioceptive, (iii) robot, and (iv) VR. For the purposes of analysis and reporting, we used the operational definitions as laid out in the introduction. Additionally, biofeedback and physical stimulation treatment groups were combined under the proprioceptive group as they exert their treatment effects with the same principle of increasing the level of proprioceptive input to the patient.^32^, ^33^, ^34^, ^35^, ^36^ Exergaming studies were subsumed under the VR treatment group if they had elements of immersive or nonimmersive VR (e.g., Xbox Kinect). We conducted Markov Chain Monte Carlo simulations using vague priors^37^ and a generalized linear model with Gaussian family distribution and an identity link function.^38^ The setting of 10,000 burn‐ins, 100,000 iterations, and 1,000 adaptations was used when conducting the analysis. Trace and density plots were used to assess for model convergence and consistency. Deviance information criterion and individual datapoint posterior mean deviance contribution were used to compare goodness of fit between the consistency and inconsistency models.^38^ The deviance information criterion was also used to select between a fixed‐effects or random‐effects model.^38^ The random‐effects model was ultimately chosen for all outcomes as it minimized the deviance information criterion. The output of the network analysis was presented as a heat plot, in which a blue cell indicates a positive value and a yellow cell indicates a negative value. For TUG, UPDRS (motor outcome), and QoL, a negative value indicates an improvement in the outcome (favoring the treatment arm). For balance score, 10MWT, and stride length, a positive value indicates an improvement in the outcome measure (favoring the treatment arm). Surface under the cumulative ranking (SUCRA) scores are presented for each outcome, ranking the interventions in terms of effectiveness, with higher SUCRA scores reflecting a higher likelihood that the treatment is most beneficial.

### Risk‐of‐bias assessment

The revised Cochrane Risk‐of‐Bias tool for randomized trials (ROB2) was used to evaluate for risk of bias in the included studies. The ROB2 tool evaluates bias across 5 features: (i) the randomization process, (ii) deviations from intended interventions, (iii) missing outcome data, (iv) measurement of the outcome, and (v) selection of the reported result. Two independent and blinded authors (ECKH and NYO) assessed all included studies for risk‐of‐bias (Supplementary Material – Table S4), and disagreements were resolved through discussion with a third independent author.

### Publication bias

Publication bias was assessed via visually inspecting for funnel plot asymmetry, using the R packages *netmeta* and *dmetar*. Funnel plots are presented in Supplementary Material – Figures S1–S6. Funnel plots were largely symmetrical throughout the different outcomes.

## Results

### Summary of included studies

**Figure 1:**
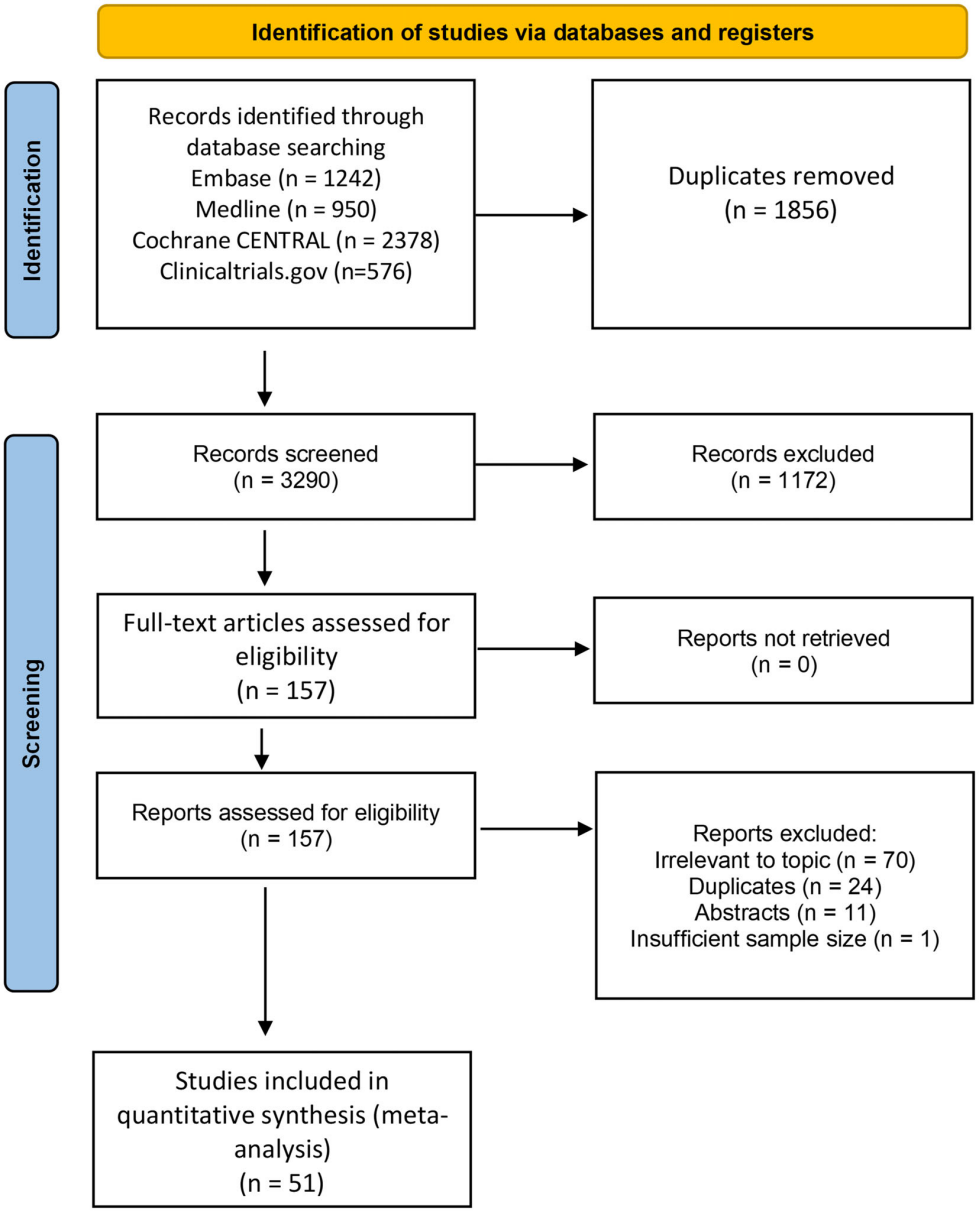
Preferred Reporting Items for Systematic Reviews and Meta‐Analyses flow diagram.

A total of 5146 studies were identified after the search in the four databases. After de‐duplication, 1289 studies were screened. Full‐text reviews were done for 157 studies, and eventually, 51 randomized controlled trials were included in this network meta‐analysis, involving 2095 patients were included in the final analysis. Six Internet‐based,^39^, ^40^, ^41^, ^42^, ^43^, ^44^ 14 proprioceptive,^18^, ^35^, ^36^, ^45^, ^46^, ^47^, ^48^, ^49^, ^50^, ^51^, ^52^, ^53^, ^54^, ^55^ 12 robotic,^17^, ^27^, ^56^, ^57^, ^58^, ^59^, ^60^, ^61^, ^62^, ^63^, ^64^, ^65^ and 19 VR^22^, ^66^, ^67^, ^68^, ^69^, ^70^, ^71^, ^72^, ^73^, ^74^, ^75^, ^76^, ^77^, ^78^, ^79^, ^80^, ^81^, ^82^, ^83^ studies were included (Fig. 1). For most of the studies, the treatment effects were measured without the technology (i.e., motor and QoL outcomes were measured when the patients were not receiving the intervention at the point of evaluation).

All controls for the studies, excluding the proprioceptive studies, were either usual care or active controls. For the proprioceptive studies, controls were placebos.

The six most reported outcomes were changes in UPDRS (motor outcome) score (as measured by UPDRS‐III or MDS‐UPDRS‐III), stride length, 10MWT, TUG test, balance scale scores (e.g., Mini Balance Evaluation Systems Test scores and Parkinson Disease Questionnaire Scores), and Quality of Life scores (e.g., SF‐36 and PDQ‐39). A summary of the included trials is included in Tables S2 and S3. The mean age of included patients ranged from 53.5 to 76.1 years for the intervention arms and 53.5 to 77.7 years for the control arms. Total duration of intervention and the duration of follow‐up/ last time of clinical assessment are reported in Table S3. Majority of the interventions lasted between 4 and 12 weeks, with follow‐up duration lasting between 4 and 12 weeks. Majority of included participants in the control and intervention arms had their functional ability rated between Hoehn and Yahr Scale Stages II and III.

### Network analysis of interventions

For all interventions, differences in effect sizes are presented. SUCRA scores are presented in Table 1.

**Table 1:** Surface under the cumulative ranking scores for respective outcomes.

	Control	Exercise	Internet‐based	Proprioceptive	Robotic	Virtual reality
UPDRS	31.75	36.87	42.53	36.26	72.95	79.63
TUG timing	4.87	46.03		43.66	75.93	79.51
Stride length	8.99	51.17		75.17	45.87	68.79
10MWT speed	14.23	43.05	51.44	66.01	77.78	47.51
Balance scale scores	15.37	43.26	45.03	40.66	68.66	87
Quality‐of‐life measures	5.64	76.79	27.9	36.73	62.69	90.24

UPDRS, Unified Parkinson's Disease Rating Scale; TUG, Timed up and go.

### UPDRS (motor component)

**Figure 2:**
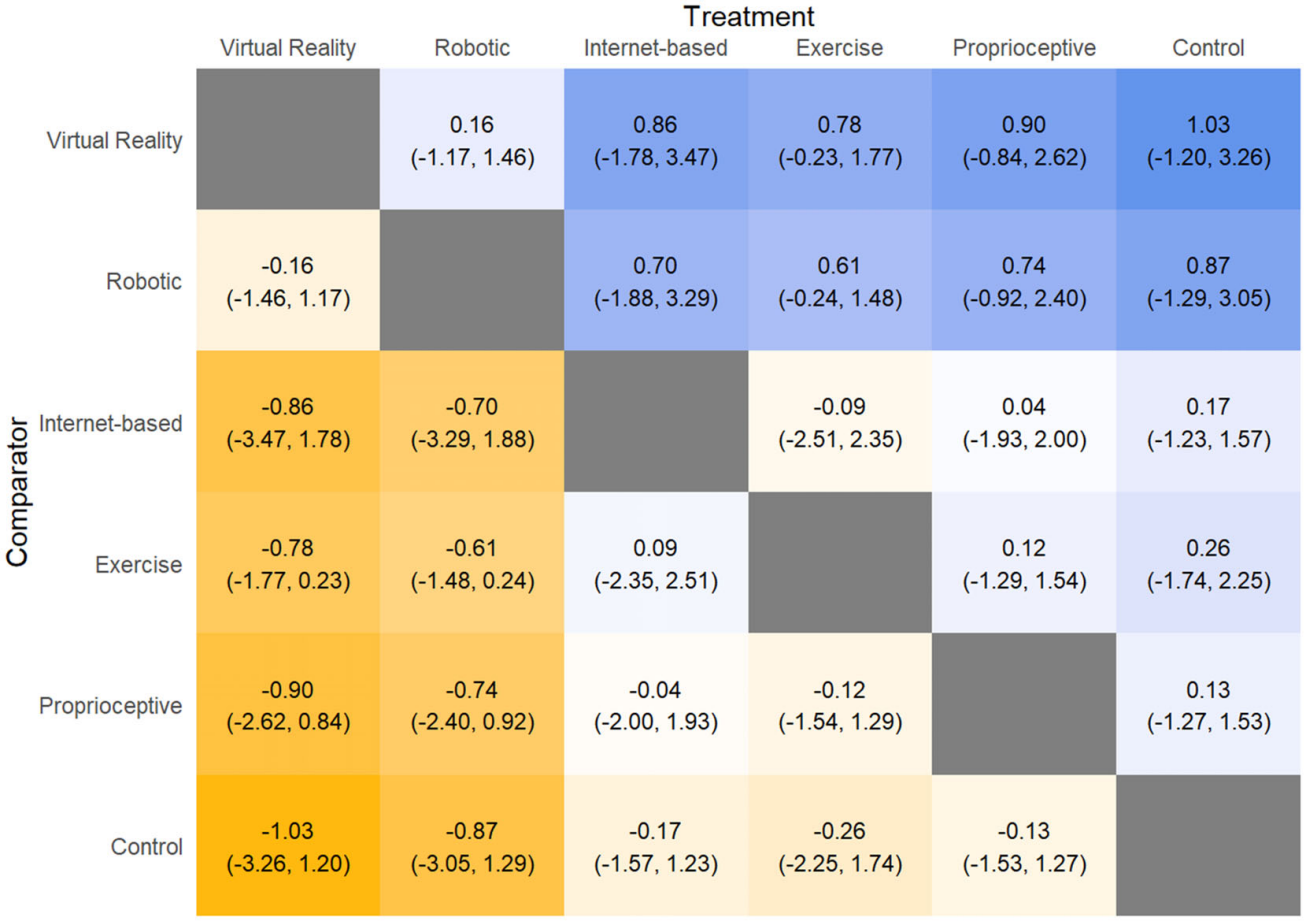
League table heat plot comparing changes in Unified Parkinson's Disease Rating Scale scores. A blue cell indicates a positive value, and a yellow cell indicates a negative value. A negative value indicates an improvement in the outcome (favoring the treatment arm).

All interventions performed similarly for UPDRS (motor component) (Fig. 2). Comparing SUCRA scores, VR (SUCRA: 79.63) performed the best, followed by robotic (SUCRA: 72.95), Internet‐based (SUCRA: 42.53), exercise (SUCRA: 36.87), proprioceptive intervention (SUCRA: 36.26), and control (SUCRA: 31.75).

### TUG

**Figure 3:**
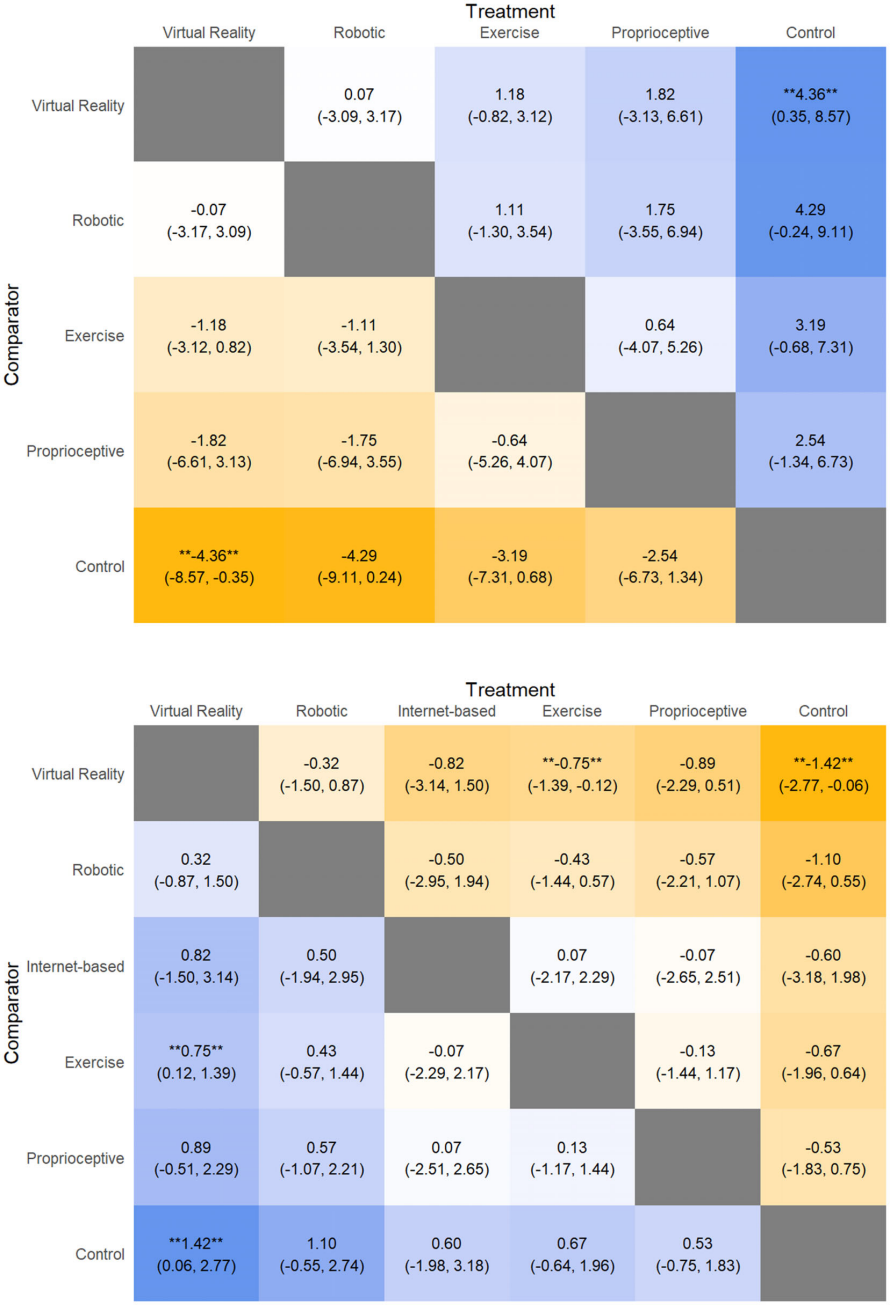
League table heat plot comparing changes in timed up‐and‐go test timings (above) and balance scale scores (below). A blue cell indicates a positive value, and a yellow cell indicates a negative value. A negative value (above) and a positive value (below) indicates an improvement in the outcome (favoring the treatment arm).

Virtual reality intervention significantly outperformed control interventions (MD: −4.36, 95% CrI: −8.57, −0.35) (Fig. 3). Comparing SUCRA scores, VR (SUCRA: 79.51) performed the best, followed by robotic (SUCRA: 75.93), exercise (SUCRA: 46.03), proprioceptive intervention (SUCRA: 43.66), and control (SUCRA: 4.87).

### Stride length

**Figure 4:**
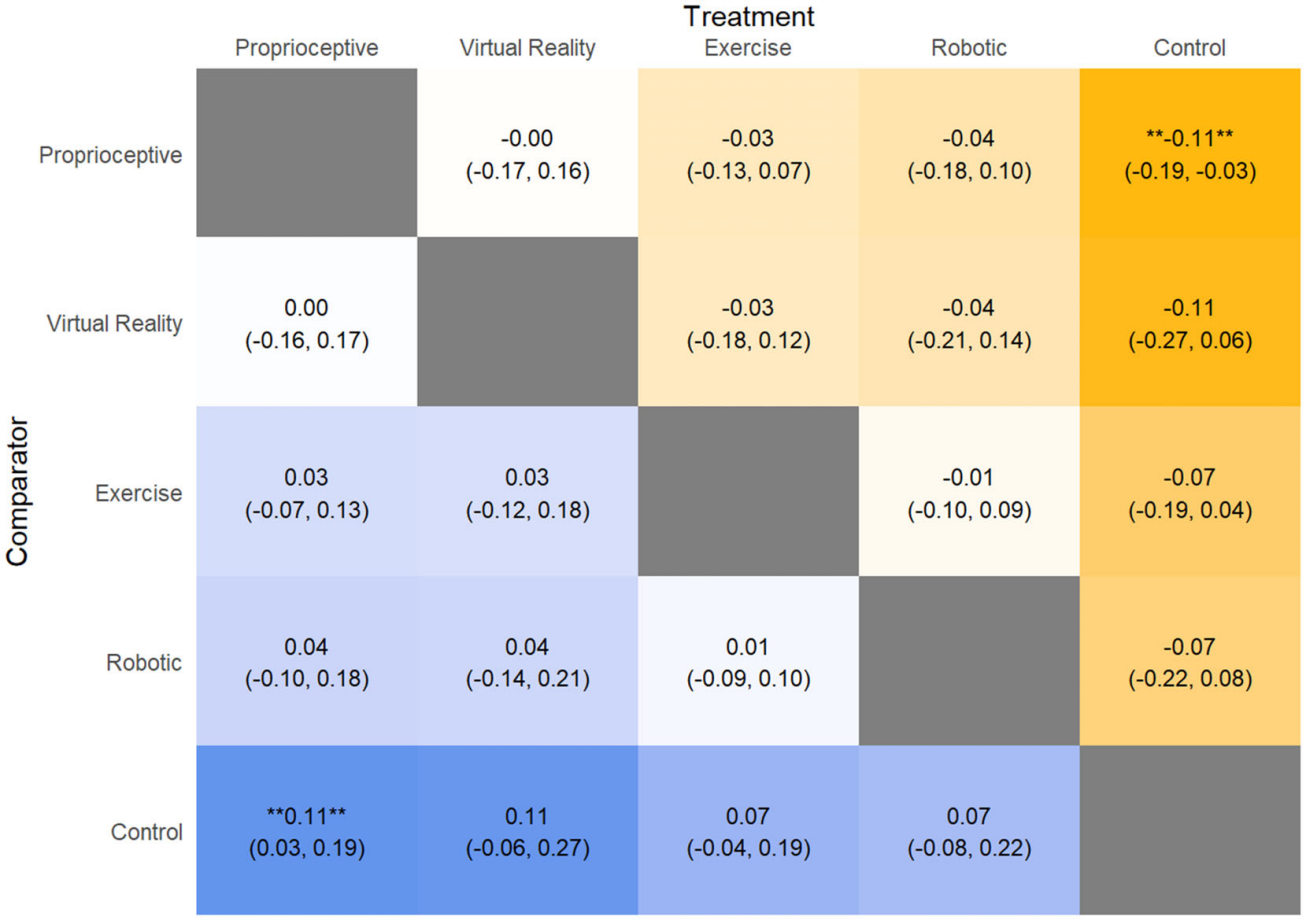
League table heat plot comparing changes in stride length. A blue cell indicates a positive value, and a yellow cell indicates a negative value. A positive value indicates an improvement in the outcome (favoring the treatment arm).

Proprioceptive intervention significantly improved stride length (Fig. 4) compared to control intervention (MD: 0.11 m, 95% CrI: 0.03, 0.19). Comparing SUCRA scores, proprioceptive intervention (SUCRA: 75.17) performed the best, followed by VR (SUCRA: 68.79), exercise (SUCRA: 51.17), robotic (SUCRA: 45.87), and control (SUCRA: 8.99).

### 10‐meter walk test

**Figure 5:**
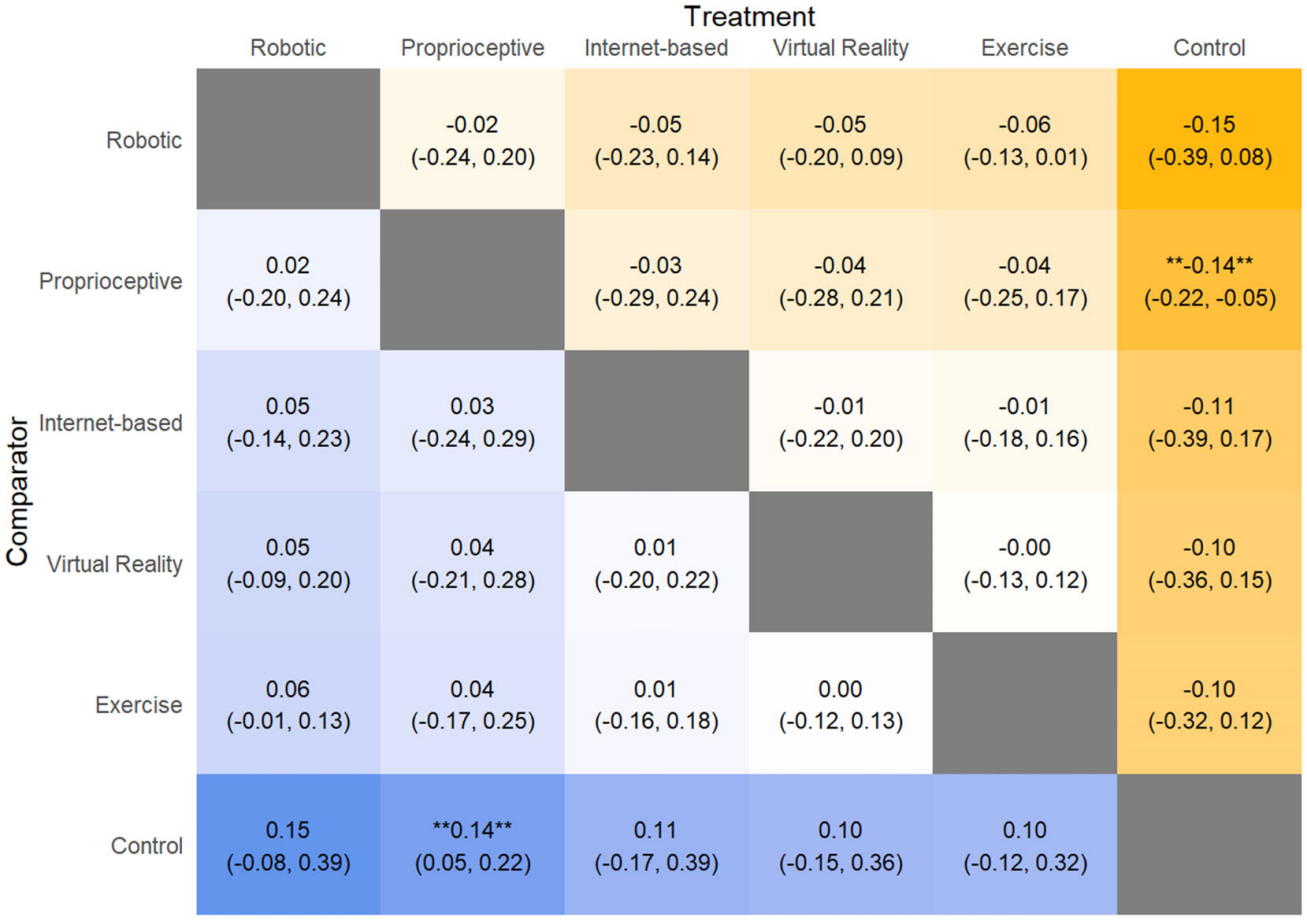
League table heat plot comparing changes in 10‐meter walk test. A blue cell indicates a positive value, and a yellow cell indicates a negative value. A positive value indicates an improvement in the outcome (favoring the treatment arm).

Proprioceptive intervention significantly improved 10MWT speed (Fig. 5) compared to control interventions (MD: 0.14 m/s, 95% CrI: 0.05, 0.22). Comparing SUCRA scores, robotic intervention (SUCRA: 77.78) performed the best, followed by proprioceptive intervention (SUCRA: 66.01), Internet‐based intervention (SUCRA: 51.44), VR (SUCRA: 47.51), exercise (SUCRA: 43.05), and control (SUCRA: 14.23).

### Balance scale scores

Virtual reality improved balance scale scores significantly (Fig. 3) compared to exercise intervention (SMD: 0.75, 95% CrI: 0.12, 1.39) and control intervention (SMD: 1.42, 95% CrI: 0.06, 2.77). Comparing SUCRA scores, VR (SUCRA: 87.00) performed the best, followed by robotic intervention (SUCRA: 68.66), then Internet‐based intervention (SUCRA: 45.03), exercise (SUCRA: 43.26), and control (SUCRA: 15.37). Two studies by Picelli et al.^57^, ^58^ reporting on balance scale scores had potentially overlapping patient cohorts. A sensitivity analysis was hence performed by preserving the study with a greater sample size.^57^ The effect sizes and SUCRA score ranking remained consistent, with VR outperforming other modalities.

### QoL scores

**Figure 6:**
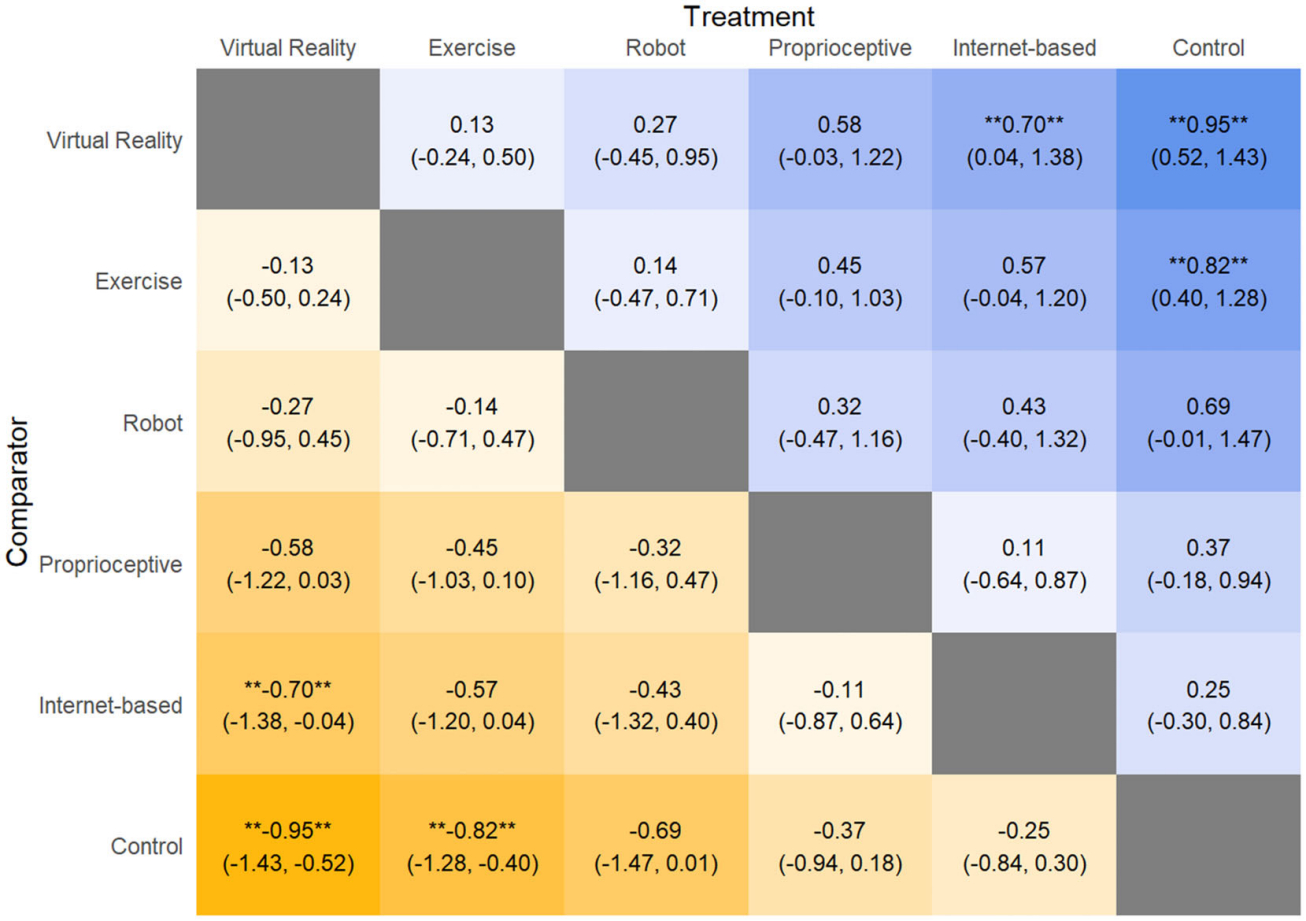
League table heat plot comparing changes in quality‐of‐life measures. A blue cell indicates a positive value, and a yellow cell indicates a negative value. A negative value indicates an improvement in the outcome (favoring the treatment arm).

Virtual reality intervention improved QoL scores (Fig. 6) significantly compared to Internet‐based interventions (SMD: −0.70, 95% CrI: −1.38, −0.04) and control interventions (SMD: −0.95, 95% CrI: −1.43, −0.52). Exercise intervention also improved QoL scores significantly compared to control intervention (SMD: −0.82, 95% CrI: −1.28, −0.40). Comparing SUCRA scores, VR intervention (SUCRA: 90.24) performed the best, followed by exercise (SUCRA: 76.79), then robotic intervention (SUCRA: 62.69), proprioceptive intervention (SUCRA: 36.73), Internet‐based intervention (SUCRA: 27.9), and control (SUCRA: 5.64).

### Quality assessment

Risk‐of‐bias assessment using the ROB2 tool is provided in Table S4. Most of the studies are at low to moderate risk of bias. For the studies with moderate risk of bias, this was largely due to the impossibility of blinding the patients to the intervention that they receive in most studies (e.g., robotic intervention), hence causing the second domain of the ROB2 tool to be flagged as “Some concerns.”

## Discussion

This network meta‐analysis of 2095 patients investigated the role of four technology‐based interventions ((i) Internet‐based, (ii) proprioceptive, (iii) robot, and (iv) VR) in improving the physical rehabilitation and quality of life of patients afflicted with PD. Our findings highlighted that the different interventions are well‐suited for improving different aspects of motor function.

Virtual reality intervention, through mediums such as Xbox Kinect or full audio‐visual sensory immersion, fares the best in terms of improving overall motor function as measured by the UPDRS motor outcome. Robotic intervention improves 10MWT results the most as measured by the SUCRA score. Virtual reality intervention improves TUG scores,^84^ a measure of functional mobility and fall risk, and balance scale scores. Several interventions improve mobility to differing degrees as measured by the outcome measures stride length and 10 MWT. Virtual reality intervention improved QoL the most significantly. While there have been previous meta‐analyses investigating the effects of exercise, VR, Internet‐based, and robotic interventions on PD patients, there has been no network meta‐analysis that compares these interventions directly, nor has there been any form of meta‐analysis aggregating the effects of proprioceptive interventions such as peripheral stimulation.

The highly significant improvements to TUG scores and balance scale scores after VR intervention are supported by existing literature, which indicate that VR intervention improves dynamic balance.^85^, ^86^ Balance is achieved and sustained through a complex influx of sensory inputs, involving different modalities (e.g., vestibular, audio, and vision). These inputs are integrated, and they interact with motor planning to finally give a motor output. It is hypothesized that VR rehabilitation most significantly improves balance as it is a global intervention that stimulates many senses at once and hence, when integrated, improve balance tremendously.^67^ PD patients are twice as likely to have falls and fractures as non‐PD subjects.^87^ Thus, restoring balance and postural stability is vital.

The improvement to 10MWT results after robotic intervention could be explained through a variety of mechanisms. It is hypothesized that robotic gait training improves gait parameters and walking capacity by facilitating repetitive gait‐like movements, synchronizing the walking pattern and strengthening the neuronal circuits contributing to gait pacing.^59^ Proprioceptive interventions via biofeedback and peripheral stimulation improve the amount of proprioceptive sensory input, which has been demonstrated to play a vital part in the production and coordination of movements.^88^ Proprioceptive interventions through pressure or vibratory stimulation may be crucial in patients in the most advanced stages of the disease. In that stage, patients may be severely compromised cognitively and can no longer undergo treatments which involve explicit learning strategies (e.g., cueing and decomposition of movements).^47^

Current therapeutic options such as pharmacotherapy and deep brains stimulation have proven sub‐optimal in improving motor outcomes such as postural abnormality. Pharmacological interventions can even aggravate nonmotor parkinsonian symptoms such as hallucinations and orthostatic hypotension.

In terms of cost, Internet‐based interventions have been shown to be largely cost‐effective in various settings,^89^ and with increased Internet connectivity, it is expected to be increasingly utilized at low cost. Carpino et al.^90^ have demonstrated that robotic interventions using operational machines such as the ones studied in the included studies^57^, ^58^, ^59^ cost the same as conventional rehabilitation. The cost‐effectiveness of VR rehabilitation is still being investigated, with studies estimating that VR incurs extra cost, but this increase may be counterbalanced when time for therapist supervision is reduced.^91^ For proprioceptive interventions, few studies have estimated their cost‐effectiveness. However, with 3D printing, it is predicted that interventions utilizing plantar stimulation will have greatly reduced cost.^92^ Safety profiles of these interventions are nascent, but preliminary findings for VR interventions indicate that it is feasible and safe.^93^ As for robotic interventions, ensuring safety is a great challenge. A mismatch in the positioning of the patient and the machine may cause unintended, unsafe interaction forces at the patient's joints.^94^ In terms of wearable robots, translation from controlled laboratory settings to uncontrolled environments such as a patient's home proves challenging.^95^

Conventional rehabilitation is considered an adjuvant to medical interventions, such as medications and surgeries, in PD treatment as it can attenuate the symptoms or even delay the disease progression.^96^ This network meta‐analysis has given credence to the idea that a neurorehabilitation program, utilizing a mix of interventions that most effectively target and improve various QoL, gait, and balance parameters, would be ideal. It should be cautioned that with the relative nascence of the field, provider experience is limited. Despite this, and the possibly high start‐up cost of setting up such programs, more research should be done utilizing these interventions and observing how these programs can be safely implemented at a low cost and monitored virtually. More cohort studies should be done to combine these approaches with traditional pharmacological approaches and establish the long‐term effects of these interventions on QoL, gait, and balance parameters of PD patients.

### Strength and limitations

This network meta‐analysis is the first to compare four technology‐based interventions: (i) Internet‐based, (ii) proprioceptive, (iii) robot, and (iv) VR. The results provide information on how the four interventions fare against active and inactive controls. The recent safety and cost‐effectiveness studies suggest that these four interventions may be implemented at scale. However, more thorough safety protocols when designing and testing the interventions are required. It is noteworthy that with increasing Internet connectivity, reduced cost of 3D printed proprioceptive interventions and highly portable VR setups, a home‐based rehabilitative approach might be viable for PD, further increasing their convenience and reducing barriers to healthcare access. However, the reliability of this study is affected by a few factors: (i) There are few nonproprioceptive studies that utilize inactive controls (ii) included studies of the same intervention do not always use the same set of outcome measures, reducing the amount of data points that can be aggregated per outcome (iii) heterogeneity was introduced due to the different final timepoints and follow‐up durations for the included studies. In some studies, it was noticed that the effects of the interventions subside after a longer time‐period, causing effect sizes to be insignificant. More cohort studies can be done to investigate the changes in effect size with time (iv) patients were aware of the intervention that they received in most studies, and this could have led them to adopt behaviors (health‐related or otherwise) that could influence the relatively subjective measurements of QoL. Future studies should track longitudinally if the effects of these technology‐based interventions persist after the intervention has stopped. Future studies can investigate if there exists a synergistic effect between conventional therapies and the technology‐based interventions outlined here. Lastly, future work should include studies on the impact of various technology‐based interventions on cognitive outcomes, in view of the potential of integrated motor‐cognitive methods for PD rehabilitation.

### Conclusion

This Bayesian network meta‐analysis demonstrated that four technology‐based interventions (Internet‐based, proprioceptive, robotic, and VR) can significantly improve QoL, gait, and balance parameters in PD patients, with each intervention being the most effective in different outcome measures. The results suggest that with current technology, a multi‐modality rehabilitative approach using a combination of conventional therapies (e.g., pharmacotherapy) and the four technology‐based interventions should be explored. With the proliferation of telemedicine technology, perhaps future studies can also investigate implementing these approaches at home to improve outcomes while increasing patient convenience and satisfaction. Research looking into the longer‐term effects of these technology‐based interventions and their interactions with conventional therapies can facilitate individualized management specific to different motor subtypes of PD.

## Funding Information

E‐K Tan is supported by the National Medical Research Council (STaR and PD LCG 000207, SPARK II Programme).

## Conflict of Interest

The authors do not have any competing financial interests or personal relationships that could have appeared to influence the work reported in this paper.

## Author Contributions

CE Yau, ECK Ho, NY Ong, CJK Loh, AS Mai, and Professor E‐K Tan contributed to (1) the conception and design of this project; (2) acquisition, analysis, and interpretation of data; (3) drafting and revising it critically for important intellectual content. All authors gave their final approval of the version to be published and agree to be accountable for all aspects of the work in ensuring that questions related to the accuracy or integrity of any part of the work are appropriately investigated and resolved.

## Supporting information


Appendix S1.

## Data Availability

Data sharing is not applicable to this article as no new data were created or analyzed in this study.
