# Supplementary material for: Innovative technology‐based interventions in Parkinson's disease: A systematic review and meta‐analysis
Source: Ann Clin Transl Neurol. 2024 Sep 5;11(10):2548–62. doi: 10.1002/acn3.52160 (PMC11514937; doi:10.1002/acn3.52160)

# Supplementary Material 1: PRISMA Checklist

| **Section and Topic** | **Item #** | **Checklist item** | **Location where item is reported** |
| --- | --- | --- | --- |
| **TITLE** | | |  |
| Title | 1 | Identify the report as a systematic review. | Pg 1 |
| **ABSTRACT** | | |  |
| Abstract | 2 | See the PRISMA 2020 for Abstracts checklist. | Pg 2,3 |
| **INTRODUCTION** | | |  |
| Rationale | 3 | Describe the rationale for the review in the context of existing knowledge. | Pg 4-6 |
| Objectives | 4 | Provide an explicit statement of the objective(s) or question(s) the review addresses. | Pg 4-6 |
| **METHODS** | | |  |
| Eligibility criteria | 5 | Specify the inclusion and exclusion criteria for the review and how studies were grouped for the syntheses. | Pg 7 |
| Information sources | 6 | Specify all databases, registers, websites, organisations, reference lists and other sources searched or consulted to identify studies. Specify the date when each source was last searched or consulted. | Pg 7 |
| Search strategy | 7 | Present the full search strategies for all databases, registers and websites, including any filters and limits used. | Table S1 |
| Selection process | 8 | Specify the methods used to decide whether a study met the inclusion criteria of the review, including how many reviewers screened each record and each report retrieved, whether they worked independently, and if applicable, details of automation tools used in the process. | Pg 7-8 |
| Data collection process | 9 | Specify the methods used to collect data from reports, including how many reviewers collected data from each report, whether they worked independently, any processes for obtaining or confirming data from study investigators, and if applicable, details of automation tools used in the process. | Pg 7-8 |
| Data items | 10a | List and define all outcomes for which data were sought. Specify whether all results that were compatible with each outcome domain in each study were sought (e.g. for all measures, time points, analyses), and if not, the methods used to decide which results to collect. | Pg 7-8 |
|  | 10b | List and define all other variables for which data were sought (e.g. participant and intervention characteristics, funding sources). Describe any assumptions made about any missing or unclear information. | Pg 7-8 |
| Study risk of bias assessment | 11 | Specify the methods used to assess risk of bias in the included studies, including details of the tool(s) used, how many reviewers assessed each study and whether they worked independently, and if applicable, details of automation tools used in the process. | Pg 9 |
| Effect measures | 12 | Specify for each outcome the effect measure(s) (e.g. risk ratio, mean difference) used in the synthesis or presentation of results. | Pg 8, 9 |
| Synthesis methods | 13a | Describe the processes used to decide which studies were eligible for each synthesis (e.g. tabulating the study intervention characteristics and comparing against the planned groups for each synthesis (item #5)). | Pg 8-10 |
|  | 13b | Describe any methods required to prepare the data for presentation or synthesis, such as handling of missing summary statistics, or data conversions. | Pg 8-10 |
|  | 13c | Describe any methods used to tabulate or visually display results of individual studies and syntheses. | Pg 8-10 |
|  | 13d | Describe any methods used to synthesize results and provide a rationale for the choice(s). If meta-analysis was performed, describe the model(s), method(s) to identify the presence and extent of statistical heterogeneity, and software package(s) used. | Pg 8-10 |
|  | 13e | Describe any methods used to explore possible causes of heterogeneity among study results (e.g. subgroup analysis, meta-regression). | Not applicable |
|  | 13f | Describe any sensitivity analyses conducted to assess robustness of the synthesized results. | Pg 13 |
| Reporting bias assessment | 14 | Describe any methods used to assess risk of bias due to missing results in a synthesis (arising from reporting biases). | Pg 9, Table S2 |
| Certainty assessment | 15 | Describe any methods used to assess certainty (or confidence) in the body of evidence for an outcome. | Pg 8-10 |
| **RESULTS** | | |  |
| Study selection | 16a | Describe the results of the search and selection process, from the number of records identified in the search to the number of studies included in the review, ideally using a flow diagram. | Pg 10 |
|  | 16b | Cite studies that might appear to meet the inclusion criteria, but which were excluded, and explain why they were excluded. | Pg 10 |
| Study characteristics | 17 | Cite each included study and present its characteristics. | Table 1 |
| Risk of bias in studies | 18 | Present assessments of risk of bias for each included study. | Table S2 |
| Results of individual studies | 19 | For all outcomes, present, for each study: (a) summary statistics for each group (where appropriate) and (b) an effect estimate and its precision (e.g. confidence/credible interval), ideally using structured tables or plots. | Table 2, Figures 2-6 |
| Results of syntheses | 20a | For each synthesis, briefly summarise the characteristics and risk of bias among contributing studies. | Pg 10 |
|  | 20b | Present results of all statistical syntheses conducted. If meta-analysis was done, present for each the summary estimate and its precision (e.g. confidence/credible interval) and measures of statistical heterogeneity. If comparing groups, describe the direction of the effect. | Figure 2-6 |
|  | 20c | Present results of all investigations of possible causes of heterogeneity among study results. | Not applicable |
|  | 20d | Present results of all sensitivity analyses conducted to assess the robustness of the synthesized results. | Pg 13 |
| Reporting biases | 21 | Present assessments of risk of bias due to missing results (arising from reporting biases) for each synthesis assessed. | Not applicable |
| Certainty of evidence | 22 | Present assessments of certainty (or confidence) in the body of evidence for each outcome assessed. | Pg 10-14, Figure 2-6 |
| **DISCUSSION** | | |  |
| Discussion | 23a | Provide a general interpretation of the results in the context of other evidence. | Pg 15-17 |
|  | 23b | Discuss any limitations of the evidence included in the review. | Pg 18 |
|  | 23c | Discuss any limitations of the review processes used. | Pg 18-19 |
|  | 23d | Discuss implications of the results for practice, policy, and future research. | Pg 15-19 |
| **OTHER INFORMATION** | | |  |
| Registration and protocol | 24a | Provide registration information for the review, including register name and registration number, or state that the review was not registered. | Pg 7 |
|  | 24b | Indicate where the review protocol can be accessed, or state that a protocol was not prepared. | Pg 7 |
|  | 24c | Describe and explain any amendments to information provided at registration or in the protocol. | Not applicable |
| Support | 25 | Describe sources of financial or non-financial support for the review, and the role of the funders or sponsors in the review. | Pg 20 |
| Competing interests | 26 | Declare any competing interests of review authors. | Pg 20 |
| Availability of data, code and other materials | 27 | Report which of the following are publicly available and where they can be found: template data collection forms; data extracted from included studies; data used for all analyses; analytic code; any other materials used in the review. | Pg 20 |

# Table S1: Search strategies

Medline

| 1 | exp " Parkinson disease"/ or ("Parkinson's*" or "Parkinson disease" or "Parkinson's disease").ti,ab. |
| --- | --- |
| 2 | exp "Robotics"/ or ("robot*" and "assist*").ti,ab. |
| 3 | exp "Telemedicine"/ or exp "Cell phone"/ or exp "Smartphone"/ or exp "Mobile applications"/ or exp "Telerehabilitation"/ or exp "Internet-Based intervention"/ or exp "Decision Support Systems, Clinical"/ or exp "Decision Making, Computer-Assisted"/ or exp "medical informatics"/  or ("tele*" or "Mobile*" or "app*" or "phone*" or "mHealth" or "smart?phone*" or "smart*" or "cell?phone*" or "tablet*" or "computer*" or "eHealth" or "message*" or "internet*" or "video* " or "video?conf*" or "pda" or "personal digital assistance" or "medical informatics").ti,ab. |
| 4 | ("exergaming" or ((gam* OR gaming OR exer*) AND (virtual* OR virtual reality OR series* OR digital*)) or (virtual* adj3 gam*) or (exer* adj3 gam*) or "Xbox" or "Game*" or "exer-gaming" or "digital exercise" or "active videogame" or "Wii" or "Wii Fit" or "Kinect" or "virtual-gam*").ti,ab. |
| 5 | exp "Virtual Reality"/ or exp "Augmented Reality"/  or ((virtual* OR digital* OR computer* OR augment* OR immersive*) AND (simulation OR reality OR environment) or "mixed reality synthetic environment" or "virtual-reality*" or "augmented-reality*" or "Computer-assisted" or "computer assisted").ti,ab. |
| 6 | exp "neurofeedback"/ or exp "feedback, sensory"/ or exp " Wearable Electronic Devices "/ or exp " Accelerometry "/  or (feedback* or biofeedback* or wearable* or acceleromet* or gyroscop* or sensor* or shoe* or insole*).ti,ab. |
| 7 | exp "physical stimulation"/ or " peripheral stimulation".ti,ab. |
| 8 | exp "Physical and Rehabilitation Medicine"/ or exp "Treatment Outcome"/ or exp "Rehabilitation"/ or exp "Walking speed"/ or exp "Postural balance"/ or exp "Gait"/  or (((phys* OR motor OR function* OR mobilit*) AND (therap* OR rehab*)) OR physiotherapy* or (gait* OR balanc* OR walk* OR strid* OR coordinat* OR (activit* adj3 (dail* OR day OR life OR liv*)) OR mov* OR mobili*) AND (train* OR therap* OR rehab* OR perform*)).ti,ab.  OR ("QOL" OR "quality of life" or "quality-of-life").ti,ab.  Or ((Motor* or physical*) AND (function* or impair* or activity* or perform*)).ti,ab. |

1 AND (2 or 3 or 4 or 5 or 6 or 7) AND 8

Limit to randomised controlled trials

Embase

| 1 | 'parkinson disease'/exp OR (parkinson* NEAR/2 disease):ti,ab |
| --- | --- |
| 2 | 'robotics'/exp  Or ("robot*" and "assist*"):ti,ab |
| 3 | 'telemedicine'/exp OR 'smartphone'/exp OR 'mobile phone'/exp OR 'mobile application'/exp OR 'telerehabilitation'/exp OR 'clinical decision support system'/exp OR 'decision support system'/exp OR 'medical informatics'/exp OR 'web-based intervention'/exp OR 'video consultation'/exp  or ("tele*" or "Mobile*" or "app*" or "phone*" or "mHealth" or "smart?phone*" or "smart*" or "cell?phone*" or "tablet*" or "computer*" or "eHealth" or "message*" or "internet*" or "video* " or "video?conf*" or "pda" or "personal digital assistance" or "medical informatics"):ti,ab |
| 4 | ("exergaming" or ((gam* OR gaming OR exer*) AND (virtual* OR virtual reality OR series* OR digital*)) or (virtual* NEAR/3 gam*) or (exer* NEAR/3 gam*) or "Xbox" or "Game*" or "exer-gaming" or "digital exercise" or "active videogame" or "Wii" or "Wii Fit" or "Kinect" or "virtual-gam*"):ti,ab |
| 5 | 'virtual reality'/exp OR 'augmented reality'/exp OR 'smart-glasses'/exp OR 'augmented reality system'/exp OR 'virtual reality system'/exp OR 'clinical decision support system'/exp OR 'decision support system'/exp OR 'medical informatics'/exp OR 'web-based intervention'/exp OR 'video consultation'/exp  ((virtual* OR digital* OR computer* OR augment* OR immersive*) AND (simulation OR reality OR environment) or "mixed reality synthetic environment" or "virtual-reality*" or "augmented-reality*" or "Computer-assisted" or "computer assisted"):ti,ab |
| 6 | 'biofeedback'/exp OR ' Neurofeedback '/exp OR ' Sensory feedback '/exp OR 'Wearable computer'/exp OR 'Accelerometry'/exp or (feedback* or biofeedback* or wearable* or acceleromet* or gyroscop* or sensor* or shoe* or insole*):ti,ab |
| 7 | 'physical stimulation'/exp or "peripheral stimulation":ti,ab |
| 8 | 'rehabilitation medicine'/exp OR 'treatment outcome'/exp OR 'rehabilitation'/exp OR 'walking speed'/exp OR 'body equilibrium'/exp OR 'gait'/exp OR 'clinical outcome'/exp OR 'patient-reported outcome'/exp  or (((phys* OR motor OR function* OR mobilit*) AND (therap* OR rehab*)) OR physiotherapy* or (gait* OR balanc* OR walk* OR strid* OR coordinat* OR (activit* adj3 (dail* OR day OR life OR liv*)) OR mov* OR mobili*) AND (train* OR therap* OR rehab* OR perform*)):ti,ab  OR ("QOL" OR "quality of life" or "quality-of-life"):ti,ab  Or ((Motor* or physical*) AND (function* or impair* or activity* or perform*)):ti,ab |

1 AND (2 or 3 or 4 or 5 or 6 or 7) AND 8

Limit to randomised controlled trials

Clinicaltrials.gov

| Condition/disease | Parkinson disease OR Parkinson's disease OR Parkinsonism OR Parkinson syndrome OR Parkinson’s syndrome |
| --- | --- |
| Intervention/treatment | Robotics OR Robot OR Robot assistance OR Telemedicine OR Cell phone OR Smartphone OR mobile application OR mobile phone OR Mobile phone application OR Telerehabilitation OR Internet based intervention OR Decision support systems OR Decision making computer assisted OR Medical informatics OR eHealth OR Video conference OR Personal digital assistance OR pda OR Medical informatics OR exergaming OR gaming OR virtual reality OR VR gaming OR virtual OR series OR digital OR Xbox OR Game OR digital exercise OR active videogaming OR active videogame OR Wii OR Wii fit OR Kinect OR virtual gaming OR Augmented reality OR immersive OR simulation OR reality OR environment OR mixed reality synthetic environment OR computer assisted OR Neurofeedback OR feedback sensory OR wearable electronic devices OR accelerometry OR feedback OR neurofeedback OR acceleromet OR gyroscop OR shoe OR insole OR physical stimulation OR peripheral stimulation OR physical rehabilitation medicine |

Cochrane

| 1 | ((Parkinson* syndrome) OR (Parkinson* NEAR syndrome) OR ("Parkinsonism") OR (Parkinson* disease) OR (Parkinson* NEAR disease) OR (Parkinson* NEXT syndrome) OR (Parkinson* NEXT disease)):ti,ab |
| --- | --- |
| 2 | ((Robot*) AND (assist*)):ti,ab |
| 3 | (("telemedicine") OR ("Smartphone") OR ("Mobile phone") OR (Mobile device*) OR ("Mobile application") OR ("telerehabilitation") OR ("clinical decision support system") OR ("decision support system") OR (“medical informatics”) OR (“web-based intervention”) OR ("video-consultation") OR ("virtual consultation") OR (Tele*) OR (Mobile*) OR (application) OR (phone*) OR ("mHealth") OR (smart?phone) OR (smart*) OR (cell?phone) OR (tablet*) OR (computer*) OR ("eHealth") OR (message*) OR (internet*) OR (video*) OR (video?conf*) OR ("pda") OR ("personal digital assistance")):ti,ab |
| 4 | (((("exergaming") OR (gam* OR exer* OR "gaming")) AND (virtual* OR "virtual reality" OR series* OR digital*)) OR (virtual* NEAR gam*) OR (exer* NEAR gam*) OR ("Xbox") OR (game*) OR (exer-gaming) OR ("digital exercise") OR (digit* exercis*) OR ("active videogame") OR ("wii") OR ("wii fit") OR ("kinect") OR (virtual-gam*)):ti,ab |
| 5 | (("virtual reality") OR ("augmented reality") OR ("smart glasses") OR ("augmented reality system") OR ("virtual reality system") OR ("clinical decision support system") OR ("decision support system") OR ("medical informatics") OR ("web-based intervention") OR ("video consultation") OR ((virtual* OR digital* OR computer* OR augment* OR immersive*) AND ("simulation" OR "reality" OR "environment")) OR ("mixed reality synthetic environment") OR ("virtual reality") OR (augmented-reality*) OR ("computer assisted")):ti,ab |
| 6 | (("biofeedback") OR ("neurofeedback") OR ("sensory-feedback") OR ("wearable computer") OR ("Accelerometry") OR (feedback*) OR (biofeedback*) OR (wearable*) OR (acceleromet*) OR (gyroscop*) OR (sensor*) OR (shoe*) OR (insole*)):ti,ab |
| 7 | (("physical stimulation") OR ("peripheral stimulation")):ti,ab |
| 8 | (("rehabilitation medicine") OR (rehab* med*) OR ("treatment outcome") OR (rehab*) OR ("walking speed") OR ("body equilibrium") OR ("gait") OR ("clinical outcome") OR ("patient-reported outcome") OR (((phys* OR motor OR function* OR mobilit*) AND (therap* OR rehab*)) OR physiotherapy* OR (gait* OR balanc* OR walk* OR strid* OR coordinat* OR (activit* NEAR/3 (dail* OR "day" OR "life" OR liv*)) OR mov* OR mobili*) AND (train* OR therap* OR rehab* OR perform*)) OR ("QOL") OR ("quality of life")):ti,ab |

1 AND (2 or 3 or 4 or 5 or 6 or 7) AND 8

Limit to randomised controlled trials

# Table S2: Baseline characteristics of patients in included studies

| Author | **Type of intervention** | **Elaboration of intervention** | **Control** | **Elaboration of control** | **Number of patients in intervention arm** | **Number of patients in control arm** | **Mean age in intervention arm** | **Mean age in control arm** | **Number of females in intervention arm** | **Number of females in control arm** |
| --- | --- | --- | --- | --- | --- | --- | --- | --- | --- | --- |
| Kleiner et al. (2018) | Proprioceptive | Mechanical pressure stimulations delivered by metallic actuators on the feet | Placebo | Placebo stimulation | 15 | 15 | 66.47 ± 9.23 | 64.73 ± 8.75 | 3 | 6 |
| Flynn et al. (2020) | Internet-Based | Home-based exercise program monitored using telehealth | Center-based exercise | NR | 20 | 19 | 72.00 ± 7.30 | 71.00 ± 6.60 | 5 | 5 |
| Furnari et al. (2017) | Robot | Robot assisted gait training with robot attached to lower limbs, helping to shift legs on treadmill in a gait cycle | Conventional gait therapy with therapist | NR | 19 | 19 | 71.50 ± 11.70 | 77.70 ± 8.30 | 8 | 9 |
| Peppe et al. (2019) | Proprioceptive | Nano-technological device which generates High Frequency Vibration (FV) at the neck and calf | Placebo | Placebo stimulation | 20 | 20 | 61.36 ± 9.90 | 61.36 ± 9.90 | 14 | 14 |
| Picelli et al. (2012) | Robot | Robot assisted gait training using gait trainer | Conventional physiotherapy and gait training | NR | 18 | 18 | 68.10 ± 10.30 | 68.70 ± 6.20 | 8 | 12 |
| Picelli et al. (2012) | Robot | Robot assisted gait training (RAGT using the Gait-Trainer GT1) | Physical therapy and exercises | NR | 16 | 15 | (both) 68.3 | NR | 14 | NR |
| Picelli et al. (2013) | Robot | Robot assisted gait training (RAGT using the Gait-Trainer GT1) | Treadmill training | NR | 20 | 20 | 68.50 ± 10.10 | 68.80 ± 7.72 | 11 | 14 |
| Picelli et al. (2014) | Robot | Robot assisted gait training with progressive gait speed increasing and body-weight support decreasing | Balance training | NR | 33 | 33 | 68.20 ± 9.20 | 69.70 ± 7.20 | 7 | 11 |
| Lai et al. (2018) | Internet-Based | Telecoach assisted exercise involving one to one training via a telehealth system | Self-regulated exercise | NR | 10 | 10 | 63.40 ± 10.40 | 70.80 ± 7.10 | 3 | 3 |
| Beck et al. (2017) | Internet-Based | Usual care supplemented by 4 virtual visits via video conferencing from a remote specialist into patients’ homes | Usual care | NR | 97 | 98 | 65.90 ± 7.80 | 66.90 ± 8.50 | 49 | 42 |
| Ribas et al. (2017) | VR | Wii Fit games using a Nintendo® video game console with a Wii Balance Board®. The latter consists of a platform with four force transducers that generate information related to force distribution as the player displaces his center of gravity in real time. | Conventional exercise | NR | 10 | 10 | 61.70 ± 6.83 | 60.20 ± 11.29 | 6 | 6 |
| Pazzaglia et al. (2019) | VR | Perform exercises in VR setting | Physical exercise | NR | 25 | 26 | 72.00 ± 7.00 | 70.00 ± 10.00 | 7 | 9 |
| Pinto et al. (2018) | Proprioceptive | Automated peripheral stimulation was applied using a commercial medical device (Gondola) and consisted of mechanical pressure in four areas of feet | Placebo AMPS stimulation | NR | 15 | 15 | 66.47 ± 9.23 | 64.73 ± 8.75 | 3 | 6 |
| Ferraz et al. (2018) | VR | Xbox360 kinect adventure games | Functional training | NR | 20 | 22 | 67.00 ± 1.60 | 70.67 ± 7.13 | 10 | 6 |
| Volpe et al. (2014) | Proprioceptive | Wearable device that transmits mechanical vibrations at the neck and calf + physiotherapy (each patient wore 3 Equistasi devices applied over the 7th cervical vertebra and on each soleus muscle tendons.) | Placebo | Inactive device | 20 | 20 | 69.50 ± 11.17 | 69.43 ± 7.02 | 13 | 11 |
| Pelosin et al. (2019) | VR | VR + treadmill training | Conventional treadmill training | NR | 17 | 22 | 73.20 ± 3.60 | 71.90 ± 4.10 | 11 | 15 |
| Feng et al. (2019) | VR | VR balance and gait training | Conventional physical therapy | NR | 14 | 14 | 67.47 ± 4.79 | 66.93 ± 4.64 | 7 | 6 |
| Carpinella et al. (2016) | Proprioceptive | Perform tailored functional tasks using Gamepad, wearable inertial 15 sensors, provided users with real-time visual and acoustic feedback | Physical exercises with no biofeedback | NR | 17 | 20 | 73.00 ± 7.10 | 75.60 ± 8.20 | 3 | 11 |
| Pompeu et al. (2012) | VR | Wii fit games | Balance exercises | NR | 16 | 16 | NR | NR | NR | NR |
| Wilkinson et al. (2016) | Internet-Based | Telehealth visit (satellite clinic near home) | Usual in person care | NR | 15 | 17 | 76.10 ± 8.40 | 76.10 ± 7.90 | NR | NR |
| Tollar et al. (2018) | VR | Xbox360 kinect games | Stationary cycling | NR | 25 | 25 | 70.00 ± 4.69 | 70.60 ± 4.10 | NR | NR |
| Shih et al. (2016) | VR | Balance based kinect exergaming intervention | Physical balance training | NR | 10 | 10 | 67.50 ± 9.96 | 68.80 ± 9.67 | 1 | 3 |
| Capecci et al. (2019) | Robot | Robot assisted gait training | Conventional treadmill training | NR | 48 | 48 | 68.10 ± 9.80 | 67.00 ± 7.60 | 29 | 24 |
| Galli et al. (2019) | Proprioceptive | Stimulation given via a steel stick which transmits pressure and vibration at 2 points on each feet | Placebo stimulation | NR | 14 | 14 | 70.50 ± 5.99 | 65.92 ± 5.63 | NR | NR |
| Gandolfi et al. (2017) | VR | Remotely supervised in-home VR balance training | In-clinic sensory integration balance training | NR | 38 | 38 | 67.45 ± 7.18 | 69.84 ± 9.41 | 15 | 10 |
| El-Tamawy et al. (2012) | Proprioceptive | Treadmill training with vibratory stimuli applied to the feet plantar surfaces and proprioceptive neuromuscular facilitation (PNF) as well as a physiotherapy program. | Routine physiotherapy program | NR | 15 | 15 | 61.40 ± 7.28 | 63.20 ± 5.60 | NR | NR |
| Unterreiner et al. (2019) | Proprioceptive | Trained with a computer system receiving instruction and feedback on a monitor | Training by certified therapist | NR | 12 | 12 | 58.20 ± 8.88 | 53.80 ± 4.28 | NR | NR |
| Ginis et al. (2016) | Proprioceptive | A smartphone application which offered positive and corrective feedback on gait | Personalized gait advice from researcher but without the CuPiD system | NR | 20 | 18 | NR | NR | NR | NR |
| Sale et al. (2013) | Robot | Robot assisted gait training | Treadmill training | NR | 10 | 10 | 70.27 ± 9.81 | 68.42 ± 9.41 | 4 | 5 |
| Santos et al. (2019) | VR | Nintendo Wii alone | Conventional exercise | NR | 13 | 14 | 61.70 ± 7.30 | 64.50 ± 9.80 | 2 | 3 |
| Carda et al. (2012) | Robot | Robotic gait training | Conventional Treadmill Training | NR | 14 | 14 | 67.87 ± 7.05 | 66.93 ± 5.13 | NR | NR |
| Sekimoto et al. (2019) | Internet-Based | Video-based telemedicine system, delivered via a tablet | Regular visits every two months | NR | 10 | 10 | 53.50 ± 5.50 | 53.50 ± 5.50 | 3 | 3 |
| Spina et al. (2021) | Robot | Robotic balance training | Conventional balance training | NR | 11 | 11 | 68.00 ± 6.90 | 67.27 ± 4.85 | 5 | 4 |
| Ellis et al. (2019) | Internet-Based | mHealth-mediated exercise program (walking with a pedometer plus engagement in planned exercise supported by a mobile health application) | Walking with a pedometer and exercise only | NR | 26 | 25 | 64.80 ± 8.50 | 63.30 ± 10.60 | 11 | 12 |
| Yang et al. (2015) | VR | Custom-made home-based virtual reality balance training system | Conventional balance training, trained by a licensed physical therapist | NR | 11 | 12 | 72.50 ± 8.40 | 75.40 ± 6.30 | 4 | 5 |
| Liao et al. (2014) | VR | Virtual reality–based Wii Fit exercise and treadmill training | Traditional exercise and treadmill training | NR | 12 | 12 | 67.30 ± 7.10 | 65.10 ± 6.70 | 6 | 6 |
| Liao et al. (2015) | VR | Virtual Reality-Based Wii Fit Training and treadmill training | Traditional exercise and treadmill training | NR | 12 | 12 | 67.30 ± 7.10 | 65.10 ± 6.70 | 6 | 6 |
| Galli et al. (2016) | Robot | Robot assisted gait training | Treadmill training | NR | 25 | 25 | 68.80 ± 6.90 | 66.40 ± 9.70 | 11 | 13 |
| Pagnussat et al. (2018) | Proprioceptive | Medical device used to deliver effective AMPS treatment. The system consists in feet supports (left and right) with electrical motors that activate metallic stimulators having a rounded tip with diameter of 2 mm. | AMPS Sham | NR | 15 | 14 | 65.31 ± 10.04 | 64.19 ± 8.42 | 3 | 6 |
| Tollar et al. (2018) | VR | High-intensity and high-frequency agility intervention program | No physical training | NR | 35 | 20 | 67.30 ± 3.40 | 67.60 ± 4.10 | 18 | 8 |
| Subramanian et al. (2016) | Proprioceptive | Neurofeedback | Motor training | NR | 13 | 13 | 67.00 ± 9.00 | 63.00 ± 11.00 | 1 | 3 |
| Chang et al. (2019) | Proprioceptive | Tetrax biofeedback balance training | Conventional training without biofeedback | NR | 10 | 10 | 67.90 ± 11.60 | 74.90 ± 7.81 | 6 | 6 |
| Romanato et al. (2021) | Proprioceptive | AMPS Equistasi | Placebo | NR | 20 | 20 | 67.46 ± 10.27 | 67.46 ± 10.27 | 9 | 9 |
| Shen et al. (2014) | Proprioceptive | Balance and gait training with augmented feedback | Lower-limb strength training for 12 weeks | NR | 18 | 17 | 63.30 ± 8.00 | 65.30 ± 8.50 | 9 | 11 |
| Kim et al. (2022) | Robot | Robot assisted gait training | Normal gait training | NR | 22 | 22 | 68.70 ± 6.90 | 67.50 ± 9.30 | 16 | 15 |
| Pedereira et al. (2013) | VR | Nintendo wii video game | Traditional physical therapy (PT) | NR | 16 | 16 | 61.10 ± 8.20 | 66.20 ± 8.50 | 5 | 4 |
| van den Heuvel et al. (2014) | VR | Workstations consisted of a flat-panel LCD monitor connected to a PC containing a total of six, commercially available, interactive dynamic balance exercises. Movement registration using a force plate | Conventional balance training | NR | 17 | 14 | 66.30 ± 6.39 | 68.80 ± 9.68 | 5 | 8 |
| Kashif et al. (2022) | VR | Additional Virtual reality (VR) and motor imagery (MI) intervention on top of physical therapy | Routine physical therapy | NR | 22 | 22 | 63.86 ± 4.57 | 62.32 ± 4.61 | 9 | 10 |
| Kafle et al. (2021) | VR | Wii console exercise | Global exercise | NR | 30 | 30 | 72.17 ± 8.19 | 72.40 ± 6.71 | 15 | 17 |
| Goffredo et al. (2023) | VR | Non-immersive virtual reality-based telerehabilitation | At-home self-administered structured conventional motor activities | NR | 49 | 48 | 67.80 ± 6.60 | 68.20 ± 5.80 | 22 | 24 |
| Kegelmeyer et al. (2024) | Robot | Robotic-assisted gait training (RAGT) with and without the exoskeleton device (EXOD) | No interventions, no exoskeleton device (EXOD), only usual care, daily activities, and ongoing exercise regimens | NR | 23 | 22 | 72 | 72 | 10 | 2 |

NR: Not reported; NA: Not applicable;

All values expressed as mean ± standard deviation or median (interquartile range) when applicable

# Table S3: Disease characteristics of patients in included studies

| Author | **Mean disease duration in intervention arm** | **Mean disease duration in control arm** | **Mean UPDRS-III/MDS-UPDRS-III score in intervention arm** | **Mean UPDRS-III/MDS-UPDRS-III score in control arm** | **Mean HnY score (SD) in intervention arm at baseline** | **Mean HnY score (SD) in control arm at baseline** | **Freezing of gait characteristics in intervention arn** | **Freezing of gait characteristics in control arm** | **Total duration of intervention** | **Total duration of follow up / last timepoint of clinical assessments** |
| --- | --- | --- | --- | --- | --- | --- | --- | --- | --- | --- |
| Kleiner et al. (2018) | NR | NR | 24.80 ± 8.06 | 25.13 ± 10.76 | Stage I-I^1/2^: 0%, Stage II: 13%, Stage II^1/2^-IV: 87% | Stage I-I^1/2^: 13%, Stage II: 7%, Stage II^1/2^-IV: 80% | FOG-Q: 15.33 ± 4.06 | FOG-Q: 13.80 ± 4.05 | 8 sessions, across 4 weeks. | Last timepoint at 8^th^ treatment session |
| Flynn et al. (2020) | 5.20 ± 5.40 | 4.70 ± 4.50 | 30.70 ± 15.70 | 28.90 ± 14.30 | Stage 1: 6 (30%), Stage 2: 5 (25%), Stage 3: 9 (45%) | Stage 1: 5 (25%), Stage 2: 9 (45%), Stage 3: 6 (30%) | 10 (50%) | 5 (25%) | Block 1 (5 weeks) of centre-based exercise  Block 2 (5 weeks) of centre-based or home-based exercise | Last timepoint at Week 10 |
| Furnari et al. (2017) | NR | NR | 32.36 ± 15.46 | 30.15 ± 12.70 | 3.1 ± 0.9 | 2.2 ± 0.5 | NR | NR | 24 sessions, across 4 weeks. | Follow up for 12 weeks after end of treatment |
| Peppe et al. (2019) | 8.35 ± 3.60 | 8.35 ± 3.60 | NR | NR | 2.45 ± 0.50 | 2.45 ± 0.50 | NR | NR | First 8 week period, following a 4 week wash out period, the patients were switched to the second 8 week period of reverse stimulation | Last timepoint at 20 weeks |
| Picelli et al. (2012) | 6.60 ± 5.40 | 7.40 ± 6.20 | 17.33 ± 3.40 | 17.50 ± 3.89 | 2.70 ± 0.3 | 2.7 ± 0.2 | NR | NR | 12 sessions, across 4 weeks. | Follow up after 1 month |
| Picelli et al. (2012) | 7.5 | NR | 46.31 ± 6.65 | 47.20 ± 7.93 | 3.45 | 3-4 | NR | NR | 12 sessions, across 4 weeks. | Follow up after 1 month |
| Picelli et al. (2013) | 6.52 ± 5.30 | 6.99 ± 6.17 | 18.00 ± 3.71 | 17.80 ± 3.62 | NR | NR | NR | NR | 12 sessions, across 4 weeks. | Follow up after 3 months |
| Picelli et al. (2014) | 7.50 ± 5.60 | 8.30 ± 4.10 | 38.00 | 40.00 | 3.0 | 3.0 | NR | NR | 12 sessions, across 4 weeks. | Follow up after 1 month |
| Lai et al. (2018) | 6.55 ± 4.52 | 7.55 ± 4.78 | NR | NR | 2.15 ± 0.47 | 2.3 ± 0.63 | NR | NR | 24 sessions, across 8 weeks | Last timepoint at 8 weeks |
| Beck et al. (2017) | 8.30 ± 6.15 | 7.60 ± 4.90 | 29.50 ± 10.20 | 28.30 ± 9.90 | NR | NR | NR | NR | 12 months | Last timepoint at 12 months |
| Ribas et al. (2017) | 6.50 ± 4.00 | 7.00 ± 2.79 | 22.00 ± 17.63 | 20.50 ± 12.04 | 1.25 | 1.5 | NR | NR | 12 weeks | Follow up after 60 days after sessions had finished |
| Pazzaglia et al. (2019) | 7.42 ± 7.67 | 4.75 ± 4.42 | 23.00 ± 9.00 | 25.00 ± 10.00 | NR | NR | NR | NR | 6 weeks | Last timepoint at 6 weeks |
| Pinto et al. (2018) | NR | NR | 24.80 ± 8.06 | 25.13 ± 10.76 | Stage I-I^1/2^: 0%, Stage II: 13%, Stage II^1/2^-IV: 87% | Stage I-I^1/2^: 13%, Stage II: 7%, Stage II^1/2^-IV: 80% | FOG-Q: 15.33 ± 4.06 | FOG-Q: 13.80 ± 4.05 | 8 sessions | Last timepoint at 8^th^ sessions |
| Ferraz et al. (2018) | 5.00 ± 2.39 | 4.67 ± 3.17 | 32.30 ± 15.48 | 29.86 ± 12.95 | 2.50 | 2.50 | NR | NR | 8 weeks | Follow up after 60 days |
| Volpe et al. (2014) | 6.93 ± 5.43 | 6.50 ± 3.99 | NR | NR | 3.0 | 3.0 | NR | NR | 2 months | Follow up after 2 months |
| Pelosin et al. (2019) | NR | NR | 30.70 ± 9.50 | 30.60 ± 10.80 | NR | NR | NR | NR | 6 weeks | Follow up after 6 months |
| Feng et al. (2019) | 7.07 ± 1.44 | 6.60 ± 1.45 | NR | NR | 3.03 ± 0.55 | 2.97 ± 0.58 | NR | NR | 12 weeks | Last timepoint at 12 weeks |
| Carpinella et al. (2016) | 7.50 ± 3.20 | 10.30 ± 5.70 | 16.60 ± 6.80 | 22.30 ± 7.30 | 2.7 ± 0.7 | 2.9 ± 0.5 | FOG-Q: 11.3 ± 4.9 | FOG-Q: 13.1 ± 3.8 | 20 sessions | Follow up after 1 month |
| Pompeu et al. (2012) | NR | NR | 10.1 ± 3.8 | 8.9 ± 2.9 | 1.7 ± 0.5 | | NR | NR | 14 sessions, across 7 weeks | Follow up 60 days after the end of training |
|  |  |  |  |  |  |  |  |  |  |  |
| Wilkinson et al. (2016) | NR | NR | 23.40 ± 12.80 | 25.90 ± 11.80 | 2.5 ± 0.7 | 2.5 ± 0.6 | NR | NR | 12 months | Last timepoint at 12 month ± 1 month |
| Tollar et al. (2018) | 7.50 ± 1.76 | 7.50 ± 2.16 | 18.20 ± 3.85 | 18.90 ± 3.11 | 2.3 ± 0.48 | 2.4 ± 0.51 | NR | NR | 5 weeks | Last timepoint at 5 weeks |
| Shih et al. (2016) | 4.03 ± 3.74 | 5.22 ± 4.85 | NR | NR | 1.6 ± 0.84 | 1.4 ± 0.52 | NR | NR | 8 weeks | Last timepoint at 8 weeks |
| Capecci et al. (2019) | 8.90 ± 5.30 | 8.90 ± 4.30 | 22.40 ± 9.50 | 24.90 ± 16.70 | 3.0 | 3.0 | FOG-Q: 9.9 ± 7.0 | FOG-Q: 8.9 ± 6.1 | 20 sessions, across 4 weeks | Last timepoint at 4 weeks. |
| Galli et al. (2019) | 8.50 ± 1.27 | 8.00 ± 1.40 | 30.10 ± 8.40 | 30.50 ± 7.60 | 3.1 ± 0.8 | 3.0 ± 0.7 | NR | NR | 6 treatment sessions, twice a week | Last timepoint at the end of the 6^th^ session |
| Gandolfi et al. (2017) | 6.16 ± 3.81 | 7.47 ± 3.90 | 44.13 ± 24.05 | 50.76 ± 24.12 | 2.50 | 2.50 | NR | NR | 21 sessions, across 7 weeks | Follow up after 1 month |
| El-Tamawy et al. (2012) | 4.00 ± 0.90 | 3.80 ± 0.90 | 31.20 ± 4.13 | 30.20 ± 3.93 | 2-3 | 2-3 | NR | NR | 8 weeks | Last timepoint at 8 weeks |
| Unterreiner et al. (2019) | 3.20 ± 2.72 | 3.50 ± 2.58 | 30.50 ± 12.43 | 24.00 ± 14.59 | 2.1 ± 0.58 | 1.8 ± 0.71 | NR | NR | 16 treatments, across 4 weeks | Last timepoint at 4 weeks |
| Ginis et al. (2016) | NR | NR | 28.35 ± 14.77 | 33.77 ± 14.36 | 2-3 | 2-3 | NFOG-Q: 14.43 ± 7.84 | NFOG-Q: 13.70 ± 7.82 | 6 weeks | Follow up after 4 weeks |
| Sale et al. (2013) | 8.41 ± 4.99 | 8.72 ± 4.74 | 53.57 ± 14.74 | 56.17 ± 13.86 | 2.5-3.5 | 2.5-3.5 | NR | NR | 4 weeks | Last timepoint at 4 weeks |
| Santos et al. (2019) | 7.00 ± 2.80 | 6.50 ± 2.00 | NR | NR | 1.4 ± 0.6 | 1.3 ± 0.3 | NR | NR | 16 sessions, across 8 weeks | Last timepoint at 8 weeks |
| Carda et al. (2012) | 3.73 ± 2.49 | 3.73 ± 1.91 | 10.33 ± 2.38 | 10.73 ± 2.32 | 2.17 ± 0.24 | 2.23 ± 0.26 | NR | NR | 4 weeks | Follow up at 6^th^ month |
| Sekimoto et al. (2019) | 7.30 ± 6.00 | 7.30 ± 6.00 | 19.83 ± 11.61 | 20.17 ± 15.91 | 2.0 | 2.0 | NR | NR | 6 months | Last timepoint at 6 months |
| Spina et al. (2021) | 6.00 ± 1.70 | 5.00 ± 2.30 | 26.55 ± 5.77 | 26.64 ± 7.27 | 1.64 ± 0.5 | 1.72 ± 0.46 | NR | NR | 20 treatments | Follow up 1 month post treatment |
| Ellis et al. (2019) | 5.90 ± 3.50 | 3.70 ± 2.10 | 31.60 ± 10.70 | 27.60 ± 9.10 | 2.0 ± 0.3 | 2.2 ± 0.4 | NR | NR | 12 months | Last timepoint at 12 months |
| Yang et al. (2015) | 9.40 ± 3.60 | 8.30 ± 4.10 | 22.50 ± 12.10 | 21.70 ± 14.40 | 3.0 | 3.0 | NR | NR | 6 weeks | Follow up at 2 weeks |
| Liao et al. (2014) | 7.90 ± 2.70 | 6.90 ± 2.80 | NR | NR | 2.0 ± 0.7 | 2.0 ± 0.8 | NR | NR | 12 sessions, across 6 weeks | Follow up at 1^st^ month |
| Liao et al. (2015) | 7.90 ± 2.70 | 6.90 ± 2.80 | NR | NR | 2.0 ± 0.7 | 2.0 ± 0.8 | NR | NR | 12 sessions, across 6 weeks | Follow up at 1 month |
| Galli et al. (2016) | 9.9 | 8.1 | 39.33 ± 8.65 | 48.67 ± 7.86 | 1.5-3 | 2-4 | NR | NR | 20 sessions, across 4 weeks | Last timepoint at 4 weeks |
| Pagnussat et al. (2018) | NR | NR | 24.69 ± 7.80 | 25.13 ± 10.31 | H&Y (1-5) – phase OFF. 1-1.5: 0%, 2: 50%, 2.5-4: 50% | H&Y (1-5) – phase OFF. 1-1.5: 19%, 2: 37%, 2.5-4: 44% | FOG-Q: 15.69 ± 4.17 | FOG-Q: 13.31 ± 4.22 | 8 treatments | Last timepoint after 8 treatments |
| Tollar et al. (2018) | 6.70 ± 2.30 | 7.10 ± 2.80 | NR | NR | 2-3 | 2-3 | NR | NR | 15 sessions, across 3 weeks | Last timepoint at 3 weeks |
| Subramanian et al. (2016) | 4.25 ± 3.17 | 4.75 ± 2.75 | 23.30 ± 9.40 | 26.70 ± 12.60 | Stage I: 7, Stage II: 7, Stage III, 1 | Stage I: 5, Stage II: 10, Stage III: 0 | NR | NR | 4 weeks | Follow up at 10 weeks |
| Chang et al. (2019) | 3.10 ± 0.70 | 3.00 ± 0.70 | NR | NR | 2.5 ± 0.5 | 2.5 ± 0.5 | NR | NR | 10 sessions, across 2 weeks | Last timepoint at 2 weeks |
| Romanato et al. (2021) | 11.88 ± 4.89 | 11.88 ± 4.89 | 73.71 ± 22.53 | 73.71 ± 22.53 | 2.46 ± 0.51 | 2.46 ± 0.51 | NR | NR | 20 weeks; 8 weeks treatment followed by 4 weeks washout period followed by 8 weeks of treatment | Last timepoint at 20^th^ week |
| Shen et al. (2014) | 8.10 ± 4.30 | 6.60 ± 4.00 | NR | NR | 2.40 ± 0.50 | 2.50 ± 0.50 | NR | NR | 12 weeks | Follow up at 12 months |
| Kim et al. (2022) | 9.32 ± 5.82 | 8.72 ± 4.45 | 32.10 ± 11.10 | 41.10 ± 12.60 | HY scale 2.5: 8 (36%), HY scale 3.0: 14 (64%) | HY scale 2.5: 7 (32%), HY scale 3.0: 15 (68%) | NFOG-Q: 9.7 ± 9.3 | NFOG-Q: 12.7 ± 7.8 | 12 sessions, across 4 weeks | Follow up at 1 month |
| Pedereira et al. (2013) | 8.60 ± 4.60 | 7.30 ± 6.60 | NR | NR | 2.5 ± 0.6 | 2.4 ± 0.7 | NR | NR | 12 sessions, across 1 month | Follow up at 4 weeks |
| van den Heuvel et al. (2014) | 8.75 ± 7.48 | 7.60 ± 7.41 | 30.10 ± 17.38 | 27.13 ± 14.73 | 2.5 | 2.5 | NR | NR | 10 sessions, across 5 weeks | Follow up at 12 weeks |
| Kashif et al. (2022) | 6.23 ± 1.85 | 6.55 ± 1.68 | 32.45 ± 3.98 | 31.86 ± 4.62 | 2.11 ± 0.74 | 2.25 ± 0.67 | NR | NR | 12 weeks | Follow up at 16^th^ week |
| Kafle et al. (2021) | NR | NR | 30.90 ± 8.33 | 31.27 ± 7.78 | 1-2 | 1-2 | NR | NR | 14 sessions, across 7 weeks | Last timepoint at 7 weeks |
| Goffredo et al. (2023) | 4 | 5 | 35.5 ± 15.7 | 40.2 ± 19.5 | 2.0 | 2.0 | NR | NR | 30 sessions, across 6-10 weeks | Last timepoint at 6^th^ or 10^th^ weeks |
| Kegelmeyer et al. (2024) | 5 | 5 | NR | NR | Stage 1: 1 (4%), Stage 2: 18 (78%), Stage 3: 4 (17%) | Stage 1: 0 (0%), Stage 2: 18 (82%), Stage 3: 4 (18%) | NR | NR | 8 weeks | Last timepoint at 8 weeks |

NR: Not reported; NA: Not applicable; FOG-Q: Freezing of Gait Questionnaire; NFOG-Q: New Freezing of Gait Questionnaire;

All values expressed as mean ± standard deviation or median (interquartile range) when applicable

# Table S4: Risk-of-bias assessment of included trials

|  | Domain 1: Risk of bias arising from the randomization process | Domain 2: Risk of bias due to deviations from the intended interventions (effect of assignment to intervention) | Domain 3: Risk of bias due to missing outcome data | Domain 4: Risk of bias in measurement of the outcome | Domain 5: Risk of bias in selection of the reported result | Overall |
| --- | --- | --- | --- | --- | --- | --- |
| Kleiner et al. (2018) | Low | Low | Low | Low | Low | Low |
| Flynn et al. (2020) | Low | Some concerns | Low | Low | Low | Some concerns |
| Furnari et al. (2017) | Low | Some concerns | Low | Low | Low | Some concerns |
| Peppe et al. (2019) | Low | Low | Low | Low | Low | Low |
| Picelli et al. (2012) | Low | Some concerns | Low | Low | Low | Some concerns |
| Picelli et al. (2012) | Low | Some concerns | Low | Low | Low | Some concerns |
| Picelli et al. (2013) | Low | Some concerns | Low | Low | Low | Some concerns |
| Picelli et al. (2014) | Low | Some concerns | Low | Low | Low | Some concerns |
| Lai et al. (2018) | Low | Some concerns | Low | Low | Low | Some concerns |
| Beck et al. (2017) | Low | Some concerns | Low | Low | Low | Some concerns |
| G. Ribas et al. (2017) | Low | Some concerns | Low | Low | Low | Some concerns |
| Pazzaglia et al. (2019) | Low | Some concerns | Low | Low | Low | Some concerns |
| Pinto et al. (2018) | Low | Low | Low | Low | Low | Low |
| Ferraz et al. (2018) | Low | Some concerns | Low | Low | Low | Some concerns |
| Volpe et al. (2014) | Low | Low | Low | Low | Low | Low |
| Pelosin et al. (2019) | Low | Some concerns | Low | Low | Low | Some concerns |
| Feng et al. (2019) | Low | Some concerns | Low | Low | Low | Some concerns |
| Carpinella et al. (2016) | Low | Some concerns | Low | Low | Low | Some concerns |
| Pompeu et al. (2012) | Low | Some concerns | Low | Low | Low | Some concerns |
| Wilkinson et al. (2016) | Low | Some concerns | Low | Low | Low | Some concerns |
| Tollar et al. (2018) | Low | Some concerns | Low | Low | Low | Some concerns |
| Shih et al. (2016) | Low | Some concerns | Low | Low | Low | Some concerns |
| Capecci et al. (2019) | Low | Some concerns | Low | Low | Low | Some concerns |
| Galli et al. (2019) | Low | Low | Low | Low | Low | Low |
| Gandolfi et al. (2017) | Low | Some concerns | Low | Low | Low | Some concerns |
| El-Tamawy et al. (2012) | Low | Low | Low | Low | Low | Low |
| Unterreiner et al. (2019) | Low | Some concerns | Low | Low | Low | Some concerns |
| Ginis et al. (2016) | Low | Some concerns | Low | Low | Low | Some concerns |
| Sale et al. (2013) | Low | Some concerns | Low | Low | Low | Some concerns |
| Santos et al. (2019) | Low | Some concerns | Low | Low | Low | Some concerns |
| Carda et al. (2012) | Low | Some concerns | Low | Low | Low | Some concerns |
| Sekimoto et al. (2019) | Low | Some concerns | Low | Low | Low | Some concerns |
| Spina et al. (2021) | Low | Some concerns | Low | Low | Low | Some concerns |
| Ellis et al. (2019) | Low | Some concerns | Low | Low | Low | Some concerns |
| Yang et al. (2015) | Low | Some concerns | Low | Low | Low | Some concerns |
| Liao et al. (2014) | Low | Some concerns | Low | Low | Low | Some concerns |
| Liao et al. (2015) | Low | Some concerns | Low | Low | Low | Some concerns |
| Galli et al. (2016) | Low | Some concerns | Low | Low | Low | Some concerns |
| Pagnussat et al. (2018) | Low | Some concerns | Low | Low | Low | Some concerns |
| Tollar et al. (2018) | Low | Some concerns | Low | Low | Low | Some concerns |
| Subramanian et al. (2016) | Low | Some concerns | Low | Low | Low | Some concerns |
| Chang et al. (2019) | Low | Low | Low | Low | Low | Low |
| Romanato et al. (2021) | Low | Some concerns | Low | Low | Low | Some concerns |
| Shen et al. (2014) | Low | Some concerns | Low | Low | Low | Some concerns |
| Kim et al. (2022) | Low | Some concerns | Low | Low | Low | Some concerns |
| Pedereira et al. (2013) | Low | Some concerns | Low | Low | Low | Some concerns |
| van den Heuvel et al. (2014) | Low | Low | Low | Low | Low | Low |
| Kashif et al. (2022) | Low | Some concerns | Low | Low | Low | Some concerns |
| Kafle et al. (2021) | Low | Some concerns | Low | Low | Low | Some concerns |
| Goffredo et al. (2023) | Low | Some concerns | Low | Low | Low | Some concerns |
| Kegelmeyer et al. (2024) | Low | Some concerns | Low | Low | Low | Some concerns |

# Supplementary Figure 1: Funnel plot for stride length


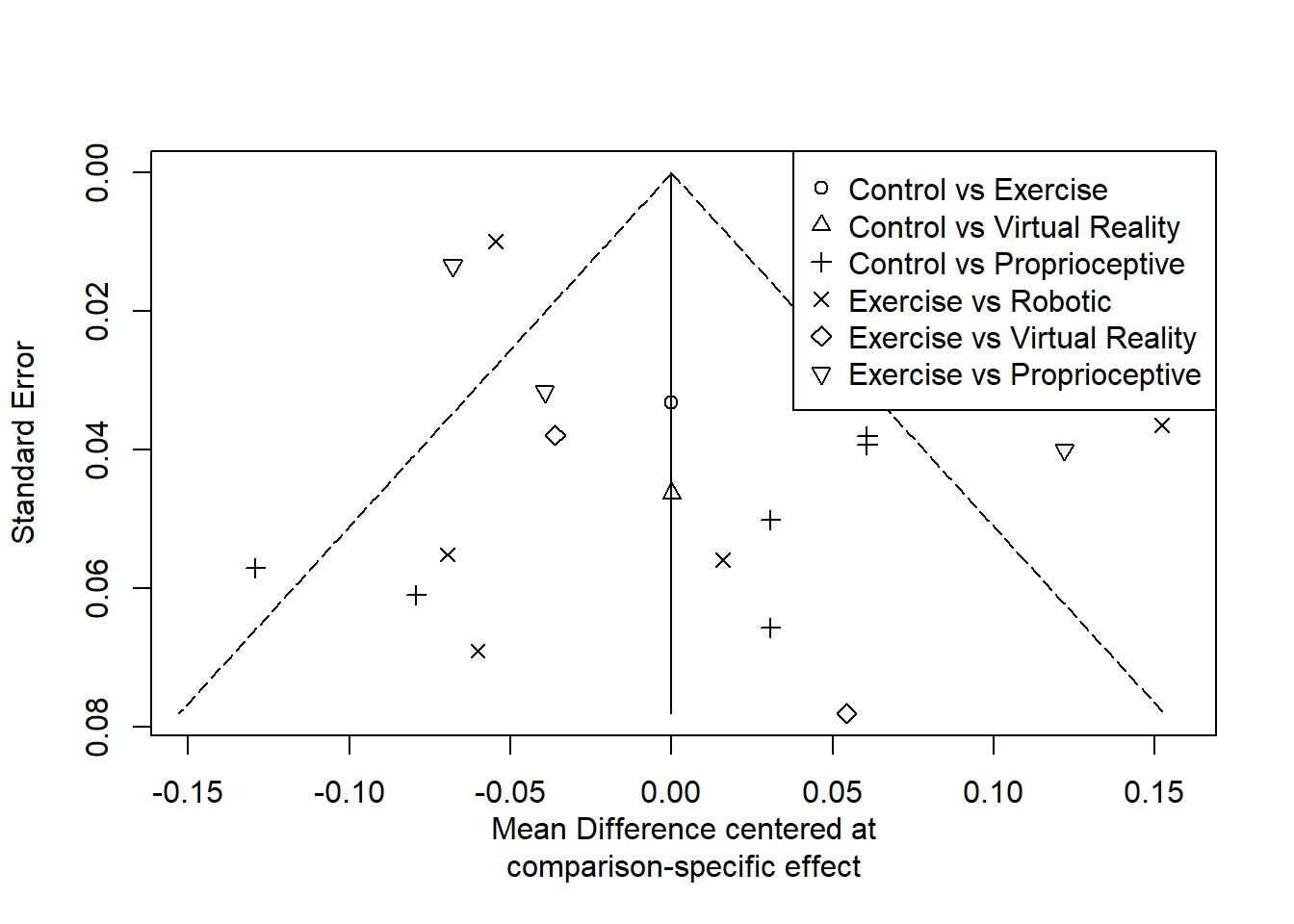


# Supplementary Figure 2: Funnel plot for 10MWT


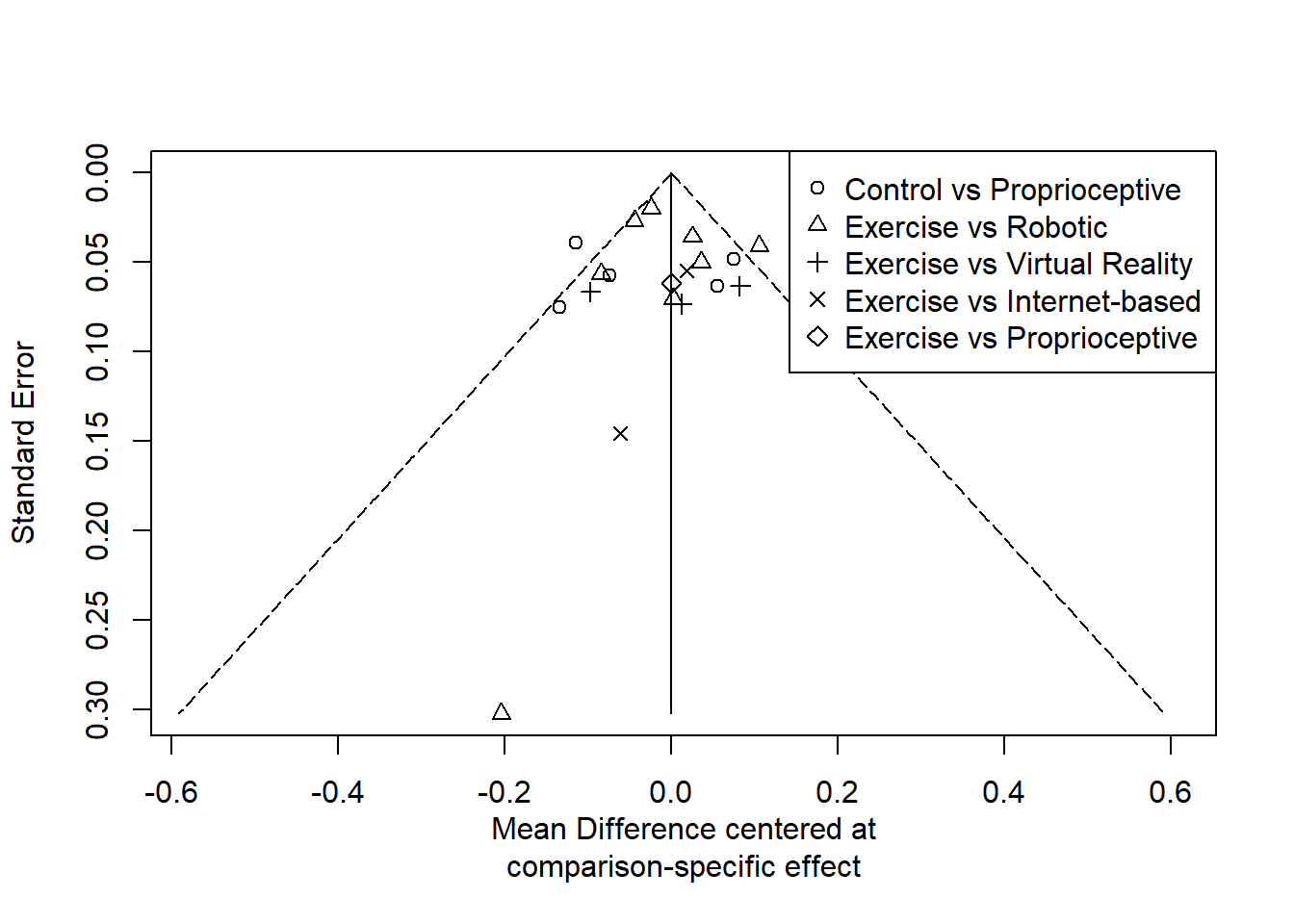


# Supplementary Figure 3: Funnel plot for TUG


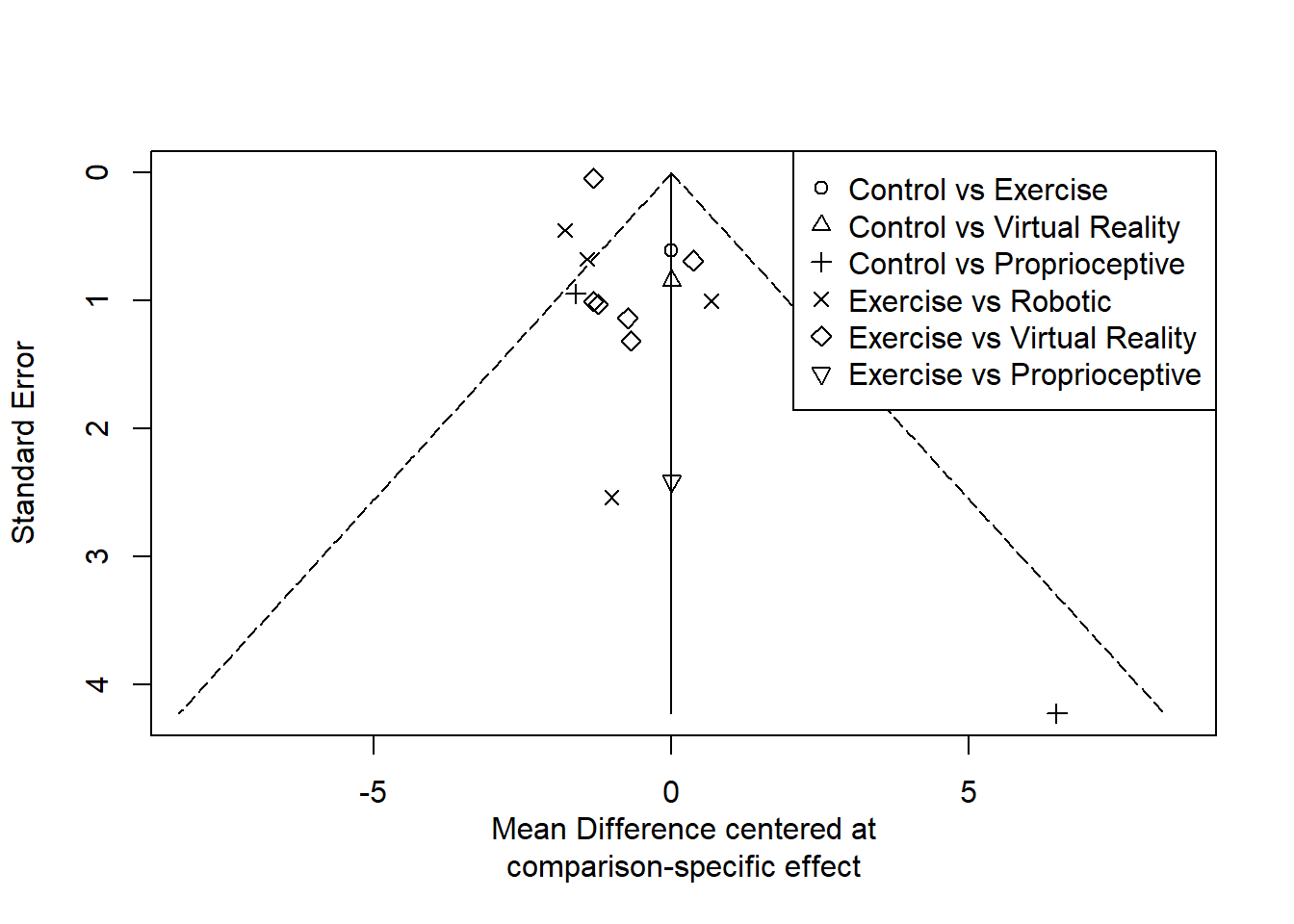


# Supplementary Figure 4: Funnel plot for UPDRS (motor outcome)


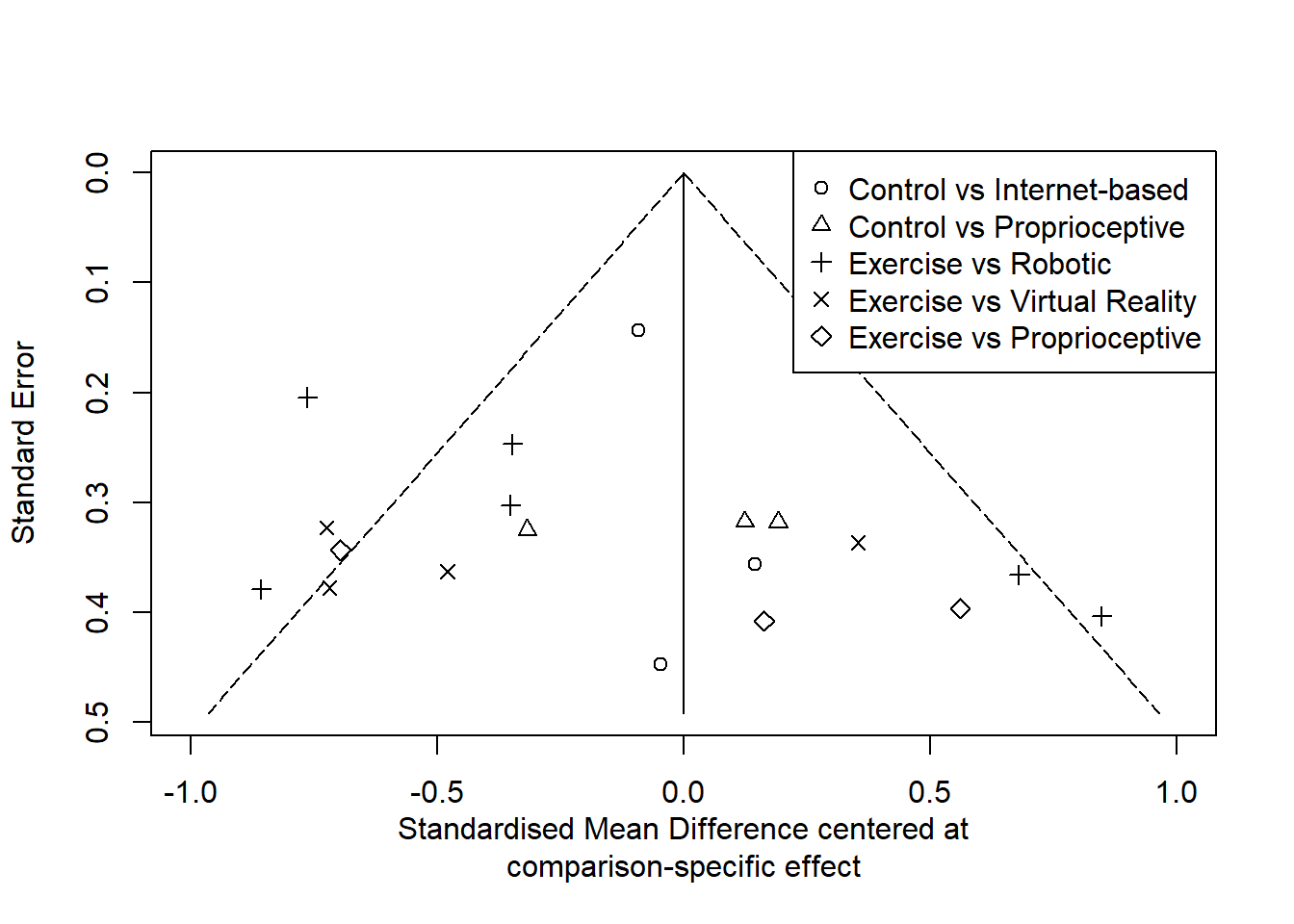


# Supplementary Figure 5: Funnel plot for balance scores


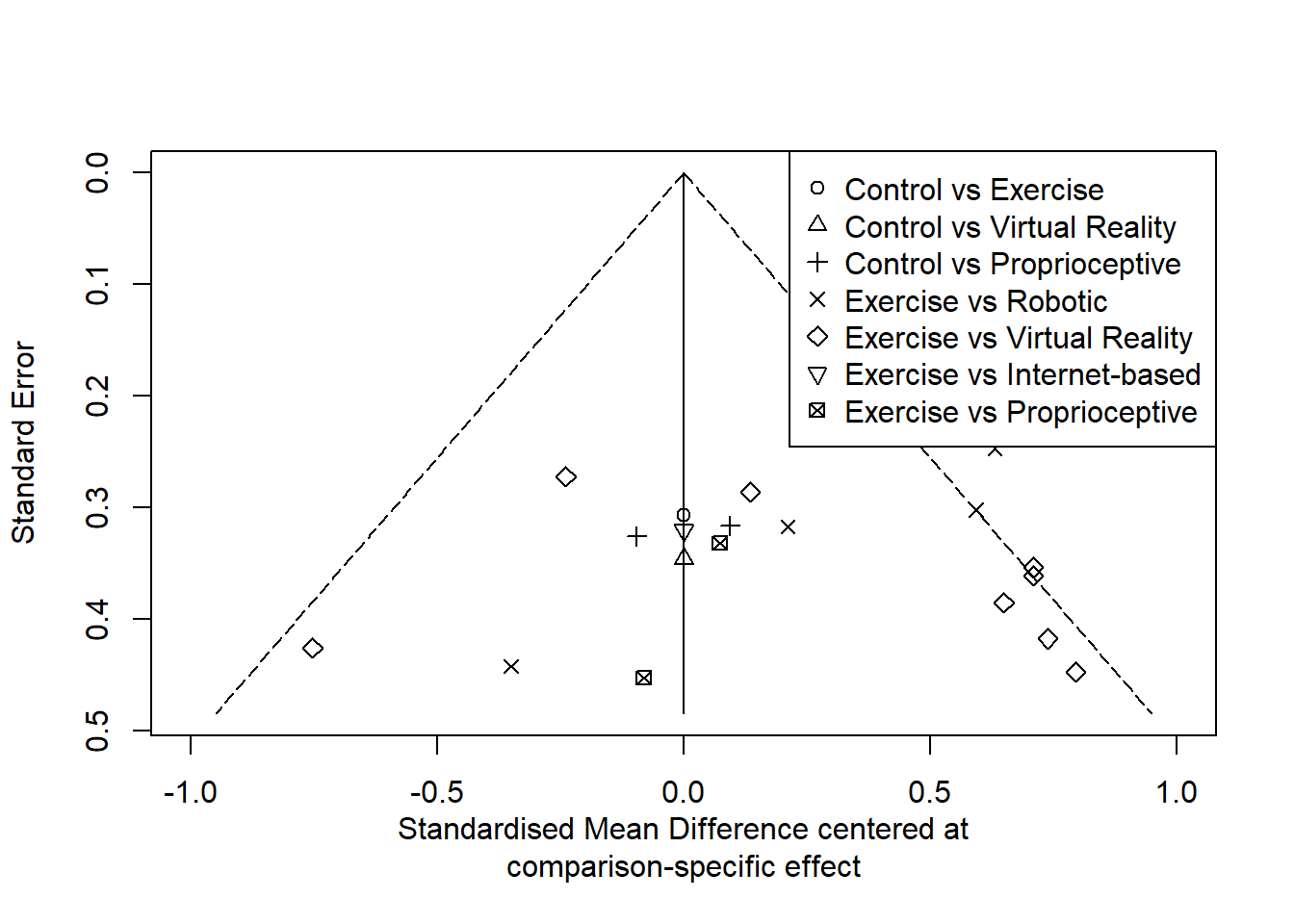


# Supplementary Figure 6: Funnel plot for QoL


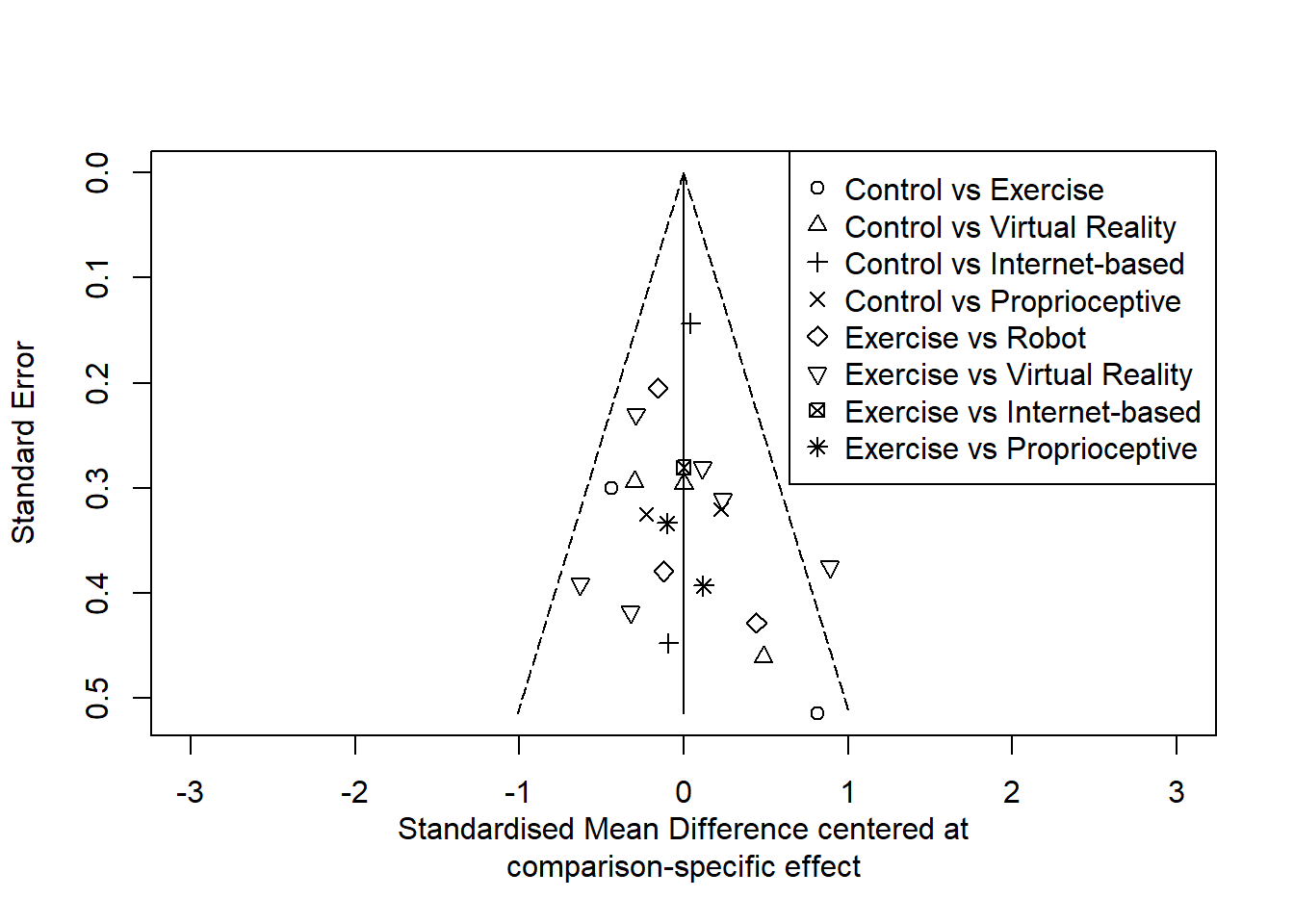

Supplement: Supplementary file 1 — Appendix S1. [file ACN3-11-2548-s001.docx]
